# Superior Fidelity and Distinct Editing Outcomes of SaCas9 Compared with SpCas9 in Genome Editing

**DOI:** 10.1016/j.gpb.2022.12.003

**Published:** 2022-12-20

**Authors:** Zhi-Xue Yang, Ya-Wen Fu, Juan-Juan Zhao, Feng Zhang, Si-Ang Li, Mei Zhao, Wei Wen, Lei Zhang, Tao Cheng, Jian-Ping Zhang, Xiao-Bing Zhang

**Affiliations:** 1 State Key Laboratory of Experimental Hematology, National Clinical Research Center for Blood Diseases, Haihe Laboratory of Cell Ecosystem, Institute of Hematology & Blood Diseases Hospital, Chinese Academy of Medical Sciences & Peking Union Medical College, Tianjin 300020, China; 2 Center for Stem Cell Medicine, Chinese Academy of Medical Sciences, Tianjin 300020, China; 3 Department of Stem Cell & Regenerative Medicine, Peking Union Medical College, Tianjin 300020, China

**Keywords:** SpCas9, SaCas9, Spacer length, Indel pattern, Knock-in efficiency, Off-target

## Abstract

A series of clustered regularly interspaced short palindromic repeats (CRISPR)-CRISPR associated protein 9 (Cas9) systems have been engineered for genome editing. The most widely used Cas9 is **SpCas9** from *Streptococcus pyogenes* and **SaCas9** from *Staphylococcus aureus*. However, a comparison of their detailed gene editing outcomes is still lacking. By characterizing the editing outcomes of 11 sites in human induced pluripotent stem cells (iPSCs) and K562 cells, we found that SaCas9 could edit the genome with greater efficiencies than SpCas9. We also compared the effects of **spacer lengths** of single-guide RNAs (sgRNAs; 18–21 nt for SpCas9 and 19–23 nt for SaCas9) and found that the optimal spacer lengths were 20 nt and 21 nt for SpCas9 and SaCas9, respectively. However, the optimal spacer length for a particular sgRNA was 18–21 nt for SpCas9 and 21–22 nt for SaCas9. Furthermore, SpCas9 exhibited a more substantial bias than SaCas9 for nonhomologous end-joining (NHEJ) +1 insertion at the fourth nucleotide upstream of the protospacer adjacent motif (PAM), indicating a characteristic of a staggered cut. Accordingly, editing with SaCas9 led to higher efficiencies of NHEJ-mediated double-stranded oligodeoxynucleotide (dsODN) insertion or homology-directed repair (HDR)-mediated adeno-associated virus serotype 6 (AAV6) donor knock-in. Finally, GUIDE-seq analysis revealed that SaCas9 exhibited significantly reduced **off-target** effects compared with SpCas9. Our work indicates the superior performance of SaCas9 to SpCas9 in transgene integration-based therapeutic gene editing and the necessity to identify the optimal spacer length to achieve desired editing results.

## Introduction

The clustered regularly interspaced short palindromic repeats (CRISPR)-CRISPR associated protein 9 (Cas9) is a powerful tool for gene editing and is widely used in basic research and clinical gene therapies [1], [2]. In this system, Cas9 endonuclease is directed by a programmable single-guide RNA (sgRNA), which is complementary to the target DNA sequence upstream of a protospacer adjacent motif (PAM) [3]. After base pairing of sgRNA and DNA, Cas9 induces DNA double-strand breaks (DSBs), which are usually repaired by nonhomologous end-joining (NHEJ), microhomology-mediated end-joining (MMEJ), or homologous recombination (HR) [4], [5]. In the presence of a homology-directed repair (HDR) template donor, precise gene knock-in or correction can be realized [6]. SpCas9 from *Streptococcus pyogenes* and SaCas9 from *Staphylococcus aureus* are the most widely used Cas9 orthologs in the CRISPR genome editing system.

The SpCas9 protein recognizes the PAM sequence of NGG, which appears every 8 bp in the genome [7]. Numerous studies have demonstrated the vigorous nuclease activity of SpCas9 in various prokaryotic and eukaryotic organisms [1], [8], [9]. However, the large size of SpCas9 (1368 amino acids, ∼ 4.1 kb) limits its further applications for adeno-associated virus (AAV)-based *in vivo* gene therapy, as the limited cargo size of the AAV vector prevents co-packaging of both SpCas9 and sgRNA expression cassettes [10]. Therefore, SpCas9 is commonly used in cell-based research and is less attractive for *in vivo* delivery. Furthermore, multiple reports have shown that SpCas9 is more likely to cause staggered breaks, leading to NHEJ-mediated error-prone DNA repair outcomes and mutations [11], [12]. Since NHEJ is highly active throughout the cell cycle in various adult cell types, many strategies have been developed to inhibit NHEJ to increase the efficiency of precise genome editing [4], [13]. As such, Cas9 orthologs with a minor bias to staggered cleavage are expected to favor HDR-mediated gene knock-in.

SaCas9 is a protein of 1053 amino acids, which is over 300 amino acids shorter than SpCas9 [14]. Benefiting from its small size, SaCas9 can be packaged with an sgRNA expression cassette in a single AAV vector. It recognizes an NNGRRT (where R is A or G) PAM, which appears every 32 bp in the genome [15]. The unique PAM pattern of sgRNA reduces the probability of SaCas9 finding suitable target sites, leading to the postulation that SaCas9 may have higher specificity than SpCas9 [16]. Indeed, several reports have shown that SaCas9 targets DNA with high specificity compared with SpCas9 [14], [17]. However, the impact of spacer lengths on its on-target and off-target effects, the indel patterns, and the HDR editing efficiency of the SaCas9-sgRNA editing system have not been comparatively investigated.

This study rigorously compared SaCas9 with SpCas9 at 11 target sites with clinical application prospects in human induced pluripotent stem cells (iPSCs) and K562 cells. We systematically investigated their nuclease activities directed by sgRNAs with different protospacer lengths (18–21 nt for SpCas9 and 19–23 nt for SaCas9). We found that SaCas9 editing was more sensitive to spacer length, with 21-nt or 22-nt sgRNA being the most effective. In addition, SaCas9 was more potent than SpCas9 when the PAM was not NNGGAT. Furthermore, for the first time, we demonstrated that SpCas9 was more prone to NHEJ +1 editing than SaCas9, thus hampering the double-stranded oligodeoxynucleotide (dsODN) insertion or AAV donor HDR integration. Finally, the GUIDE-seq analysis revealed a considerably higher fidelity of SaCas9 compared with SpCas9. Therefore, our study demonstrates that SaCas9 is a superior nuclease for manipulating human genomes and clinical gene therapy.

## Results

### Using an optimized sgRNA scaffold and a novel Cas9 fusion protein improves genome editing efficiency

**Figure 1:**
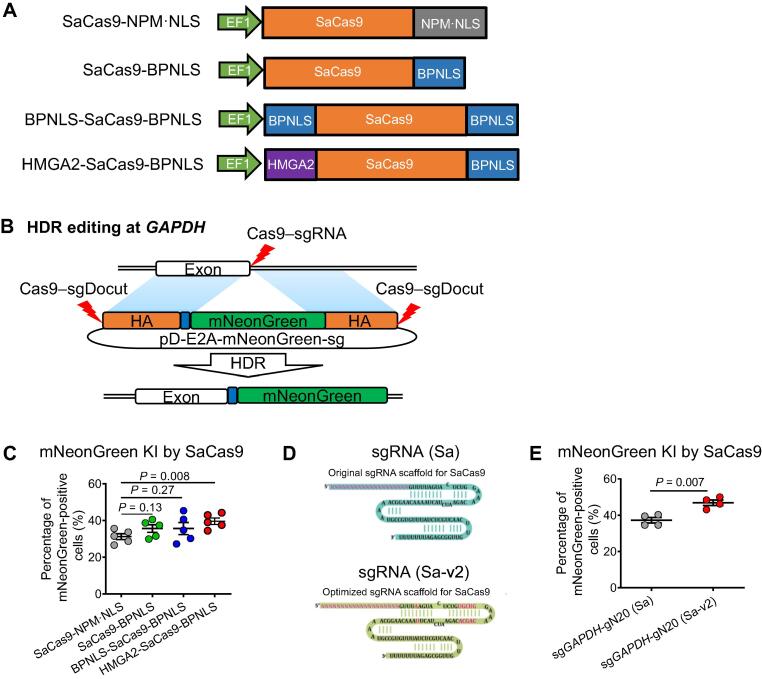
**Optimization of sgRNA structure and Cas9 NLS to improve genome editing efficiencyof SaCas9** **A.** Schematic diagram of SaCas9 variants with different NLSs. SaCas9 expression was driven by the EF1 promoter. **B.** Schematic of HDR-mediated gene editing at *GAPDH*. sgRNA was designed to target the last intron upstream of the stop codon. A promoterless double-cut HDR donor pD-E2A-mNeonGreen-sg was used to guide HDR-mediated insertion of the mNeonGreen fluorescent protein-coding gene. The orange boxes indicate the left and right HAs (both 600 bp in length); the blue box indicate a self-cleaving linker for multicistronic expression; the red lightening indicates the Cas9–sgRNA cleavage site. **C.** Cas9 fusion proteins enhance HDR editing efficiency of SaCas9. mNeonGreen-positive cells were determined by FACS 3 days after electroporation of K562 cells (*n* = 5). **D.** Scaffold optimization of sgRNA interacting with SaCas9. sgRNA (Sa) indicates the sgRNA with the original sgRNA scaffold for SaCas9; sgRNA (Sa-v2) indicates the optimized sgRNA including a U > A mutation and UGCUG addition. **E.** The scaffold-optimized sgRNA improves the HDR editing efficiency at *GAPDH* in K562 cells (*n* = 4). gN20 indicates the 21-nt sgRNA commenced with a mutant guanine to ensure the U6 ptomoter activation. Data are shown as mean ± SD. Significance (*P* < 0.05) was calculated using unpaired two-tailed Student’s *t*-test. sgRNA, single-guide RNA; NLS, nuclear localization signal; BPNLS, bipartite nuclear localization signal; HDR, homology-directed repair; HA, homologous arm; FACS, fluorescence-activated cell sorting; KI, knock-in.

Two early studies on the SaCas9-sgRNA editing system used a nuclear localization signal (NLS) from the *NPM* gene [18], [19]. We investigated whether the fusion of codon-optimized wild-type SaCas9 with other NLSs or HMGA2 (a nonhistone architectural chromatin factor) can improve its performance. Bipartite nuclear localization signal (BPNLS) can efficiently localize Cas9 to the nucleus, thus improving gene targeting efficiencies [20]. In addition, the fusion of SpCas9 with chromatin-modulating peptides derived from high-mobility group proteins improves its activity [21]. Based on these studies, we constructed multiple SaCas9 constructs (Figure 1A).

To quantitate the editing activities of these fusion SaCas9 vectors, we performed a reporter knock-in assay in K562 cells (Figure 1B). After electroporation with sg*GAPDH*, Cas9, and a double-cut donor plasmid pD-E2A-mNeonGreen-sg [22], successful HDR editing would lead the cells to fluoresce green. As expected, no mNeonGreen-positive cells were detected in the negative control that omitted sg*GAPDH* by fluorescence-activated cell sorting (FACS) 72 h after electroporation (data not shown). Consistent with early studies of SpCas9, the fusion of SaCas9 with a single BPNLS enhanced the editing efficiency by ∼ 30% compared with SaCas9 fused with NPM·NLS (Figure 1C). However, two BPNLSs at both the N- and C-termini did not further increase the HDR efficiency. Of note, an N-terminal HMGA2 and a C-terminal BPNLS additions showed significantly increased editing efficiency (Figure 1C). Therefore, we chose the HMGA2-SaCas9-BPNLS construct for further studies.

sgRNA expression was driven by the U6 RNA polymerase III promoters, for which a TTTT stretch is sufficient for transcription pause and sometimes termination, leading to reduced transcription [23], [24]. The original sgRNA scaffolds for both SpCas9 and SaCas9 start with GUUUU, and a mutation of thymine at position 4 can significantly increase the activity of low-performance sgRNA [25]. In addition, extending the duplex with UGUCG could also improve editing efficiency [26]. For SpCas9, we adopted the sgRNA scaffold with the best performance, which contains a T4 > C mutation (GUUUC) and UGUCG addition [26]. We wondered whether the optimized sgRNA (Sa-v2) could also perform better when complexed with HMGA2-SaCas9-BPNLS (Figure 1D). Consistent with a previous study, the modified sgRNA (Sa-v2) significantly increased mNeonGreen knock-in efficiencies in *GAPDH* (∼ 40%–50%; Figure 1E). We carried out the following genome editing studies using the optimized sgRNA (Sa-v2) based on the aforementioned results.

### Experimental design for comparing SpCas9 and SaCas9 editing systems

To stringently compare the cleavage efficiencies of SaCas9 and SpCas9, we designed sgRNAs targeting the sites with the NGGRRT PAM, which both SpCas9 and SaCas9 can recognize. We also constructed HMGA2-SpCas9-BPNLS to compare with HMGA2-SaCas9-BPNLS, and the TTTT stretch was mutated to prevent premature transcriptional termination. We chose 11 sites from 8 genes, including *AAVS1*, *ALB*, *PD1*, *B2M*, *CCR5*, *TRAC*, *CIITA*, and *CD326* (also known as *EPCAM*), due to their clinical potential in cell and gene therapy (Table S1).

**Figure 2:**
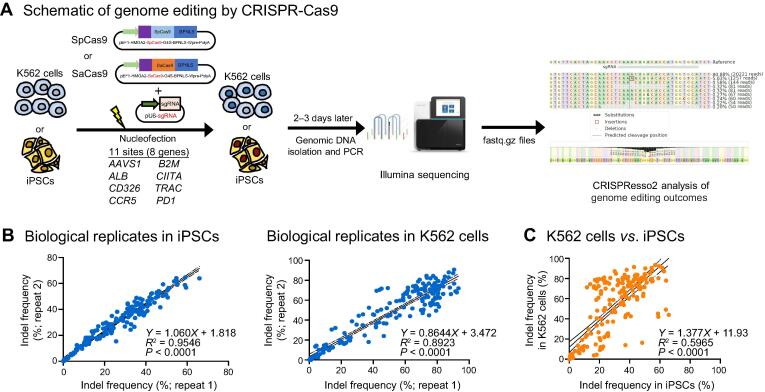
**Experimental design and data reproducibility** **A.** Schematic of genome editing with the CRISPR-Cas9 strategy in K562 cells and iPSCs. **B.** The high reproducibility of genome editing results in K562 cells and iPSCs. The indel data at 48 h and 72 h after electroporation were combined for analysis. **C.** The correlation of indel frequencies between K562 cells and iPSCs. Pearson linear regression analysis was conducted in (B) and (C). iPSC, induced pluripotent stem cell; Indel, insertion and deletion.

We carried out this study in both human iPSCs and K562 cells (a human myelogenous leukemia cell line). At 2–3 days after electroporation with Cas9–sgRNA plasmids, target loci were amplified with barcoded primers and pooled for high-throughput sequencing. To determine the indel levels, we analyzed the data using CRISPResso2 analysis (Figure 2A). We observed maximum editing 48 h after transfection, and no significant change in indel efficiency was observed 24 h later (Figure S1). Therefore, we aggregated the editing data collected at 48 h and 72 h after CRISPR delivery for analysis. Pearson correlation showed a good reproducibility of editing indel frequency in both iPSCs (*R*^2^ = 0.9546) and K562 cells (*R*^2^ = 0.8923) (Figure 2B). In addition, the activities measured in K562 cells and iPSCs were well correlated, although the overall efficiencies in K562 cells were slightly higher than those in iPSCs (Figure 2C; *R*^2^ = 0.5965; *P* < 0.0001). As such, we combined the iPSC and K562 editing data in the following sections to increase statistical power.

### The optimal spacer length for efficient cleavage with SpCas9 is 20 nt

**Figure 3:**
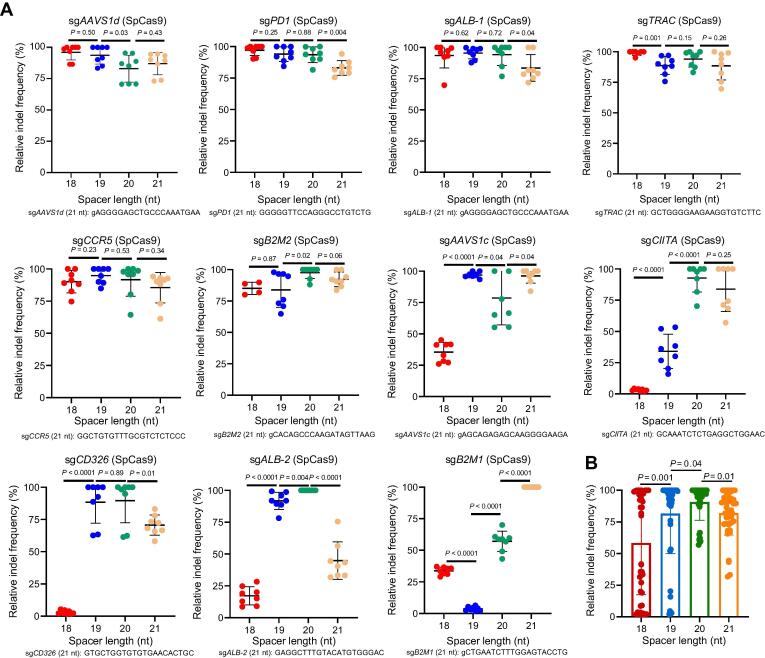
**The effectof spacer length on SpCas9 editing efficiency** **A.** Relative indel frequencies of SpCas9 in complex with sgRNAs of 18–21 nt in spacer length (*n* = 4–8 replicates for each spacer length). The indel values were normalized to the highest editing efficiency for each target. The sgRNA sequences of 21 nt are shown. **B.** Statistical analysis of relative indel frequencies of SpCas9 in complex with sgRNAs with different spacer lengths. Data are shown as mean ± SD. Significance (*P* < 0.05) was calculated using an unpaired two-tailed Student’s *t*-test.

Previous studies have compared the editing efficiencies of the truncated sgRNAs of 17–18 nt with the wild type of 20 nt and lower efficiencies were often observed in 17-nt sgRNAs [27], [28]. However, the spacer length effect has not been systematically explored. Thus, we compared 11 distinct sgRNAs with spacer lengths of 18, 19, 20, and 21 nt. All sgRNAs started with guanine (either matched G or mismatched g) to ensure U6 promoter-directed transcription [29]. The editing efficiencies of six sites showed less than 20% differences, with spacer lengths ranging from 18 nt to 21 nt (*AAVS1d*, *PD1*, *ALB-1*, *TRAC*, *CCR5*, and *B2M2*) (Figure 3A; Figure S2). However, the editing efficiencies varied with different spacer lengths at the other five sites. In four cases (*AAVS1c*, *CD326*, *CIITA*, and *ALB-2*), we observed very low efficiencies with 18-nt spacers, whereas one extra nucleotide led to a considerable increase in editing efficiency. At two sites (*B2M1* and *CIITA*), the 20-nt sgRNA showed enhanced activity compared with the 19-nt version. Further lengthening the spacer to 21 nt gave rise to varied consequences, in which two (*AAVS1c* and *B2M1*) showed increased efficiencies and four (*PD1*, *ALB-1*, *CD326*, and *ALB-2*) displayed decreased efficiencies (Figure 3A). Surprisingly, the indel efficiency of the 19-nt sgRNA was significantly lower than that of the 18-nt sgRNA at *B2M1*, which could not be explained by errors since we verified the sgRNA sequences multiple times.

At the five locations showing similar editing efficiencies between the 18-nt and 19-nt groups, we observed two with a matched G and three with a mismatched g. However, at four sites displaying lower editing efficiencies with the 18-nt spacers, we observed three with a matched G and one with a mismatched g. These data suggest that the mismatched g at the 5′ end of the spacer may affect the editing efficiency in some cases, but not necessarily, given that the matched G could also result in low efficiency.

All these aggregating data showed that 20-nt sgRNAs function better than sgRNAs with shorter or longer spacers (Figure 3B). Our data demonstrate that the optimal spacer length for SpCas9 is 20 nt, but sgRNAs with a spacer of 18 nt, 19 nt, or 21 nt may occasionally have higher activity. These results highlight the importance of testing the optimal spacer length in clinical gene therapy.

### The optimal spacer length for efficient cleavage with SaCas9 is 21–22 nt

**Figure 4:**
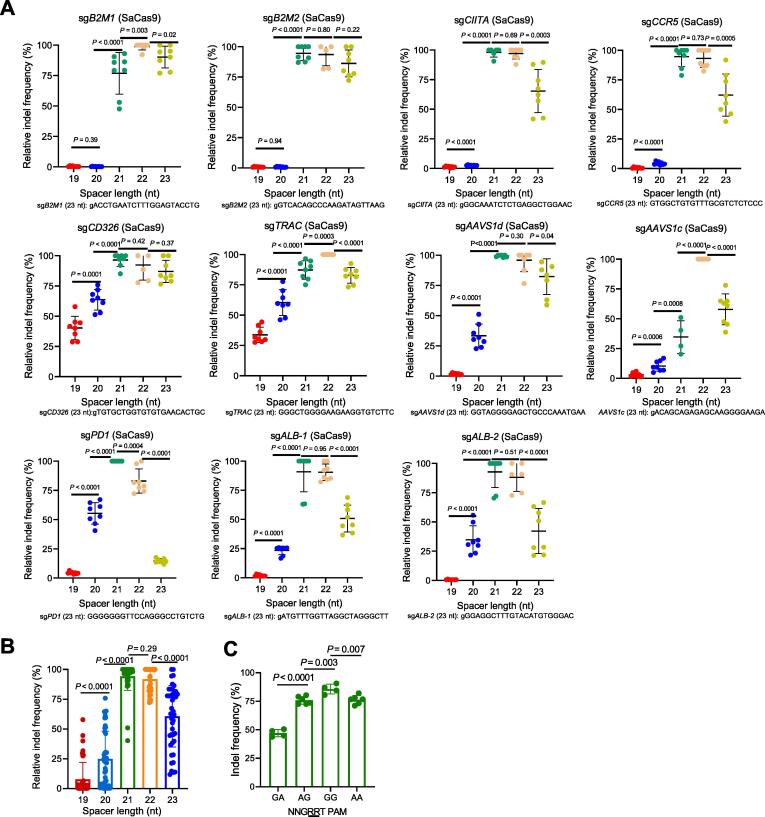
**The effectof spacer length on SaCas9 editing efficiency** **A.** Relative indel frequencies of SaCas9 in complex with sgRNAs of 19–23 nt in spacer length (*n* = 8 replicates for each spacer length). The indel values were normalized to the highest editing efficiency for each sgRNA. The sgRNA sequences of 23 nt are shown. **B.** Statistical analysis of relative indel frequencies  of SaCas9 in complex with sgRNAs with different spacer lengths. **C.** Effects of different PAMs on indel frequencies. All the sites were divided into four groups based on their PAM sequences (*n* = 4–6 replicates for each PAM sequence). Data are shown as mean ± SD. Significance (*P* < 0.05) was calculated using an unpaired two-tailed Student’s *t*-test. PAM, protospacer adjacent motif.

We then investigated the effect of spacer lengths of sgRNAs for SaCas9. Previous studies showed that the optimal spacer length for SaCas9 is 21 nt [17]; thus, we decided to compare sgRNAs of 19, 20, 21, 22, and 23 nt in length. In contrast to SpCas9, SaCas9 editing was more sensitive to spacer length. The spacer length of 19 nt showed no (9/11) or low (2/11) cleavage activity (Figure 4A; Figure S3). Extension of one nucleotide significantly increased indel frequencies, but 4/11 still showed no activity. However, further elongation of the spacer to 21 nt increased the sgRNA functionality to the highest levels, except for sg*B2M1*, sg*TRAC*, and sg*AAVS1c*, whose best activity was achieved with a 22-nt spacer. At 9 of 11 sites, extension of spacer length from 22 nt to 23 nt significantly reduced the sgRNA activity. In aggregate, the most effective sgRNAs for SaCas9 have a spacer of 21–22 nt (Figure 4B). Finally, we investigated the effects of different ‘NNGRRT’ PAMs on editing efficiency. Consistent with a previous report [15], we observed the highest indel frequency for sgRNAs that recognize targets with the ‘NNGGGT’ PAM and the lowest indel frequency for sgRNAs that recognize targets with the ‘NNGGAT’ PAM (Figure 4C).

### SaCas9 is superior to SpCas9 in generating indels

**Figure 5:**
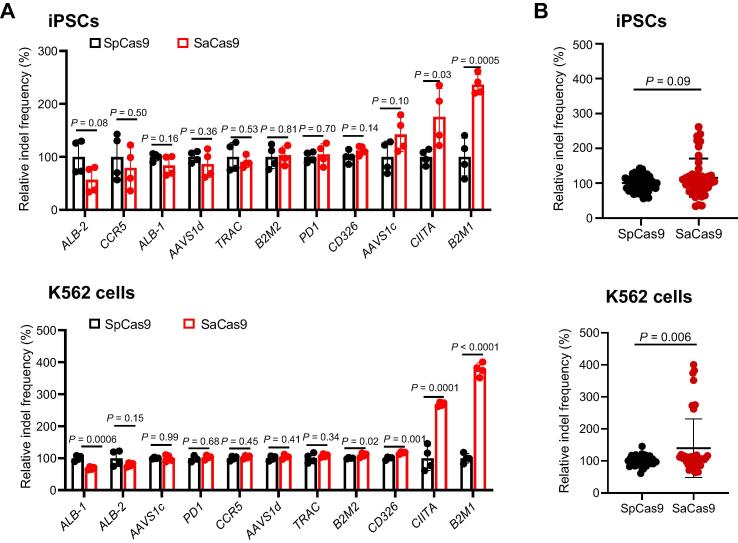
**Comparison of relative indel frequencies of SpCas9 and SaCas9** **A.** Comparison of SpCas9 and SaCas9 gene editing efficiencies at 11 individual sites in iPSCs (top) and K562 cells (bottom). **B.** Comparison of the average gene editing efficiencies of SpCas9 and SaCas9 in iPSCs (top) and K562 cells (bottom) by aggregating the data shown in (A). All the indel values were normalized to the average editing efficiency of SpCas9. Data are shown as mean ± SD. Significance (*P* < 0.05) was calculated using an unpaired two-tailed Student’s *t*-test.

We further compared the indel frequencies of SpCas9 with SaCas9 in human iPSCs and K562 cells. We chose the spacer length with the highest activity for each site for a fair comparison, which is usually 20 nt for SpCas9 and 22 nt for SaCas9. SpCas9 only showed higher relative indel frequencies at one locus in K562 cells, whereas SaCas9 displayed higher relative indel frequencies at two loci in iPSCs and four loci in K562 cells (Figure 5A). A combination of all the data for analysis showed that SaCas9 had higher cleavage activities than SpCas9 in K562 cells (*P* = 0.09 for iPSCs; *P* = 0.006 for K562 cells) (Figure 5B). We also analyzed the data by excluding the low-performance sgRNAs targeting the NNGGAT PAM and observed superior cleavage activities of SaCas9 to SpCas9 in both cell types (Figure S4). These results suggest that carefully designed sgRNAs might achieve high-level editing in the SaCas9 system.

### SaCas9 has a strikingly lower potential for staggering cleavage than SpCas9

**Figure 6:**
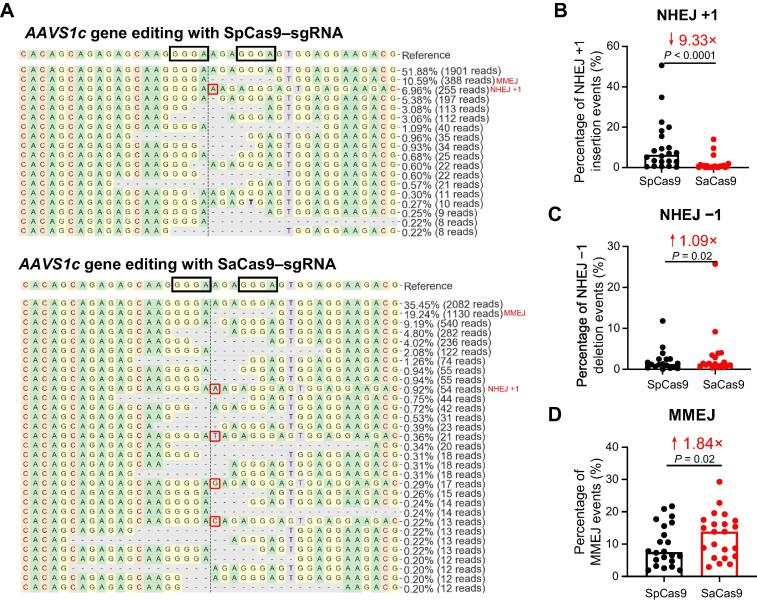
**Comparison of NHEJ +1, NHEJ −1, and MMEJ frequencies after editing with the SpCas9 and SaCas9 editing systems** **A.** Representative repair patterns after SpCas9 (top) and SaCas9 (bottom) cleavage. The microhomologies are highlighted in the black boxes. The NHEJ +1 insertion events are shown in the red boxes. All the repair patterns of the 11 sites in both iPSCs and K562 cells are displayed in Figure S5. **B.** Comparison of the percentages of NHEJ +1 insertion events between SpCas9 and SaCas9. **C.** Comparison of the percentages of NHEJ −1 deletion events between SpCas9 and SaCas9. **D.** Comparison of the percentages of MMEJ events between SpCas9 and SaCas9. Wilcoxon matched-pairs tests were conducted. *P* < 0.05 was considered statistically significant. NHEJ, nonhomologous end-joining; MMEJ, microhomology-mediated end-joining.

We observed strikingly different DSB repair patterns or outcomes after editing with SpCas9 and SaCas9. We selected the most frequent NHEJ and MMEJ alleles to conduct quantitative analysis. G deleting in G|G or C deleting in C|C or deletion between two microcolonies of 2–5 nt was designated as MMEJ (in which ‘|’ indicates the Cas9 cleavage site) [12]. The repair outcomes that cannot be interpreted as MMEJ were considered as NHEJ. The most frequent NHEJ alleles were +1 (in particular +T). In a representative example, the +A allele amounted to ∼ 7% of the total indels for SpCas9 but less than 1% for SaCas9 (Figure 6A). Statistical analysis showed that there were ∼ 10-fold fewer +1 NHEJ editing alleles in SaCas9 than in SpCas9 (Figure 6B). The reduced +1 NHEJ editing led to a slight increase in the frequencies of −1 NHEJ (such as deleting G in T|G) and MMEJ alleles in SaCas9 (*P* = 0.02; Figure 6C and D). Similarly, when the predominant +1 editing outcomes were not significantly different, the frequencies of MMEJ alleles between the two Cas9 orthologs were similar (*ALB-1* and *CIITA*; Table S1). We observed identical patterns in both iPSCs and K562 cells (Figure S5). The spacer lengths did not affect the editing outcomes (data not shown), suggesting that the distinct editing allele frequencies are a consequence of the intrinsic differences between SpCas9 and SaCas9. Since all the +1 NHEJ alleles are the duplication of the fourth nucleotide upstream of the PAM, a hallmark of repair outcome of staggered cleavage by the Cas9 endonuclease [11], we conclude that, compared with SpCas9, SaCas9 has a considerably lower tendency to generate staggered DSB ends.

### SaCas9 editing favors the NHEJ-mediated dsODN insertion and HDR-mediated gene knock-in

Our recent work on the SpCas9 system has demonstrated that +1 NHEJ is the speedy pathway to repair Cas9-mediated DSBs, which outcompetes MMEJ and HDR editing outcomes [12]. Given that SaCas9-created DSBs are less likely to be fixed by +1 NHEJ, we hypothesized that SaCas9 might be a favorable nuclease for applications such as transgene knock-in.

**Figure 7:**
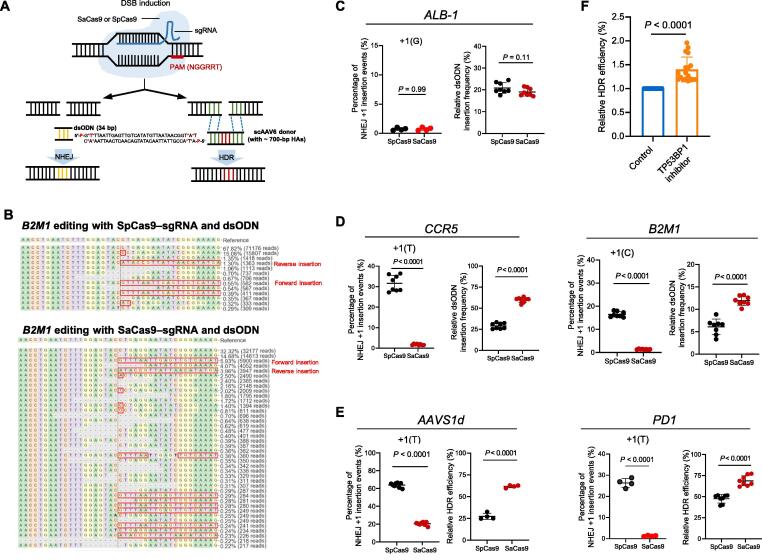
**SaCas9 editing favors NHEJ-mediated dsODN insertion and HDR-mediated geneknock-in** **A.** Schematic diagram of dsODN insertion through NHEJ and AAV donor knock-in through HDR after Cas9–sgRNA cleavage. **B.** Representative repair patterns after editing with Cas9–sgRNA and dsODN. As shown in the red boxes, dsODN could be integrated with both forward and reverse orientations. **C.** Similar NHEJ +1 frequencies between SpCas9 and SaCas9 editing at *ALB-1* are associated with similar dsODN insertion levels. **D.** Considerably lower NHEJ +1 frequencies after SaCas9 editing at *CCR5* and *B2M1* lead to a marked increase in dsODN insertion. **E.** Lower NHEJ +1 frequencies after SaCas9 editing at *AAVS1d* and *PD1* increase HDR-mediated AAV donor knock-in. **F.** The TP53BP1 inhibitor enhances HDR editing efficiency in our improved SaCas9 editing system. The relative HDR efficiency was normalized to the control (*n* = 15). Data are shown as mean ± SD. Significance (*P* < 0.05) was calculated using paired two-tailed Student’s *t*-test. dsODN, double-stranded oligodeoxynucleotide; AAV, adeno-associated virus; DSB, double-strand break.

We first used a blunt, 34-bp dsODN as a gene insertion template to investigate NHEJ-mediated gene knock-in (Figure 7A). CRISPResso2 analysis by alignments with the wild-type reference sequence showed both forward and reversed insertions of dsODN (Figure 7B). As a control, we chose *ALB-1*, for which +1 NHEJ alleles were not predominant and were similar between the two Cas9 orthologs, and observed indistinguishable relative insertion levels of dsODN between SpCas9 and SaCas9 editing (*P* > 0.05; Figure 7C). Relative editing frequency was calculated by dividing the number of single alleles by the total editing events. However, when +1(T) and +1(C) NHEJ alleles were the predominant SpCas9 editing outcomes for *CCR5* and *B2M1*, respectively, SaCas9-mediated dsODN insertion significantly increased ∼ 100% compared with the SpCas9 group (*P* < 0.0001; Figure 7D). We then compared the effects of the two orthologs on HDR-mediated gene knock-in. Adeno-associated virus serotype 6 (AAV6) has been successfully used as an HDR template in editing iPSCs and hematopoietic cells [12]. To allow for data analysis by next-generation sequencing (NGS), we designed AAV HDR donors containing an insertion of a 6-bp fragment flanked by ∼ 700-bp homologous arms (HAs) (Figure 7A). Similar to the dsODN insertion results, at sites with high-level +1(T) NHEJ tendencies, such as *AAVS1d* and *PD1*, SaCas9-mediated HDR increased ∼ 100% relative to the SpCas9 counterpart (*P* < 0.0001; Figure 7E). We conclude that SaCas9 is more conducive to NHEJ-mediated dsODN insertion and HDR editing, especially for targets with a strong tendency to form staggered ends.

Finally, we explored whether the TP53BP1 inhibitor can further improve HDR efficiency in our improved SaCas9 editing system. TP53BP1 is an essential regulator of the DSB repair pathway and functions to favor NHEJ over HDR by suppressing end resection [30]. Using a genetically encoded inhibitor of TP53BP1, we found that the precise HDR efficiency increased by ∼ 40% (Figure 7F), consistent with a previous report [30].

### GUIDE-seq revealed superior fidelity of SaCas9 to SpCas9

**Figure 8:**
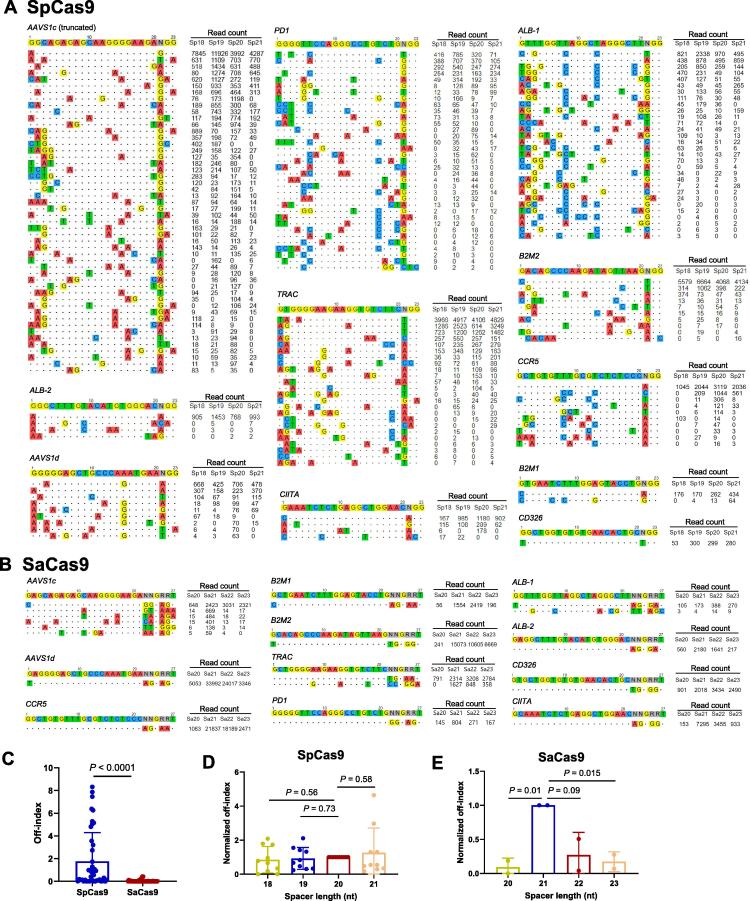
**GUIDE-seq analysis revealed higher specificity of SaCas9 than SpCas9** **A.** Off-target sites identified by GUIDE-seq for SpCas9. **B.** Off-target sites identified by GUIDE-seq for SaCas9. The target sequence with PAM is shown on the top line. Mismatches found in off-targets are highlighted in color. The read counts corresponding to different spacer lengths are shown on the right. The off-targets of *AAVS1c* (SpCas9) are truncated, and the full list is shown in Figure S6. Sp18–Sp21 indicate different spacer lengths of sgRNAs used for SpCas9-mediated editing; Sa20–Sa23 indicate different spacer lengths of sgRNAs used for SaCas9-mediated editing. **C.** Comparison of the off-index values between SpCas9 and SaCas9. The off-index was calculated as the total off-target reads divided by the on-target reads. **D.** Spacer lengths do not affect SpCas9 specificity. **E.** Effects of spacer lengths on SaCas9 specificity. The off-index values of SpCas9 and SaCas9 were normalized to 20 nt and 21 nt, respectively. Data are shown as mean ± SD (n ≥ 2). Significance (*P* < 0.05) was calculated using paired two-tailed Student’s *t*-test.

Off-targeting is an important safety concern for CRISPR-Cas9 therapeutic applications. To assess the genome-wide specificity of SaCas9 and SpCas9, we performed GUIDE-seq analysis to label the Cas9-induced breaks, followed by high-throughput sequencing and data analysis with published procedures [31]. For the 11 sgRNA target sites, we observed ∼ 350 off-target sites after SpCas9-mediated editing (Figure 8A) compared with less than 10 off-target sites after SaCas9-mediated editing (Figure 8B). Of note, 8 of 11 sites edited with SaCas9 did not show any off-target effects. These results demonstrate the superior fidelity of SaCas9.

We decided to use the off-index metric (total off-target reads divided by on-target reads) to quantitate the off-target effects. A comparison of the two editing systems showed that the off-index of SaCas9 was ∼ 20-fold lower than that of its SpCas9 counterpart (Figure 8C). For editing with SpCas9, spacer lengths from 18 nt to 21 nt did not show significant differences in the off-index (Figure 8D), suggesting that truncated sgRNAs may not be able to reduce the off/on-target ratio in SpCas9-based applications. For SaCas9, at the two sites with a substantial number of off-target reads, *AAVS1c* and *TRAC*, the 22-nt sgRNAs showed a considerably lower off-index than their 21-nt counterparts (Figure 8B and E). Together, carefully designed and prescreened 22-nt sgRNAs with SaCas9 may abrogate the off-target cleavage events.

## Discussion

SpCas9 and SaCas9 are two of the best-characterized nucleases for genome editing. Several independent studies have focused on their nuclease activities and clinical applications [14], [32]. Here, we report a novel Cas9 fusion protein with enhanced nuclease activity in mammalian cells. We systemically compared SpCas9 and SaCas9 in terms of spacer lengths, indel frequencies and patterns, knock-in efficiencies, and off-target activities using the improved design. Our results validated and extended previous reports on SaCas9 [14]. For the first time, we report that SaCas9 has a considerably lower propensity for staggered cuts, which is beneficial for dsODN insertion and HDR editing. In addition, SaCas9’s on- and off-target activities are more sensitive to the spacer length of sgRNA. We recommend using SaCas9 with sgRNA of 22 nt, either gN21 or GN21, to achieve high editing efficiency and low off-target cuts.

CRISPR-Cas9 evolved in prokaryotes. In its natural setting, Cas9 does not encounter the nuclear envelope and nucleosomes. To facilitate transfer across nuclear pores, one needs to tag Cas9 with the NLS. We and others have shown that the BPNLS from SV40 is more potent than the NLS from the *NPM* gene. Therefore, one copy of BPNLS is sufficient for efficient editing. In addition, nucleosomes impede Cas9 access to DNA [33], particularly in less accessible chromatin. Therefore, the fusion of Cas9 with a chromatin remodeler may improve its functionality in eukaryotes. Here, we report that the HMGA2-Cas9-BPNLS performs better than the commonly used Cas9 fusion protein. In addition, we adopted the modified sgRNA scaffold to prevent premature transcription termination by mutating the T4 strip and increase the binding of sgRNA and Cas9 by extending the repeat:anti-repeat duplex [26]. With all these modifications, editing efficiencies are considerably improved.

SpCas9 was the default nuclease version in most gene editing applications. Although it has been well studied, we also investigated the effects of spacer lengths in our improved system. Consistent with previous studies, we found that in many cases, spacers of 18–21 nt showed similar editing efficiencies. As a general principle, 20 nt is the optimal length for high activity, consistent with another report that extension with one nucleotide decreases its activity [34]. However, we found that the optimal spacer size was 18, 19, or 21 nt for a few sgRNAs. Therefore, we recommend screening sgRNAs of 18, 19, 20, and 21 nt for clinal applications. In support of our finding, certain 18-nt sgRNAs are more effective than the 20-nt version for SpCas9-mediated gene knockout in hematopoietic stem cells [35]. In contrast to a previous report that truncated sgRNAs showed less off-target activities, we observed similar adverse effects for sgRNA spacers ranging from 18 nt to 21 nt. These results suggest that wild-type SpCas9 is inappropriate for applications sensitive to off-target cleavage.

SaCas9 is the ideal nuclease for *in vivo* gene editing due to its small size, high efficiency, and low off-target activities. We validated previous reports that 21-nt or 22-nt sgRNAs are optimal for SaCas9 editing. We also found that the best PAM sequence was NNGGGT, followed by NNGAGT and NNGAAT. Therefore, if possible, one would avoid choosing a target site with the NNGGAT PAM. We show that SaCas9 is more effective at locations other than those bearing the NNGGAT PAM. This result is consistent with the report that SaCas9 has higher cleavage activity than SpCas9 at target sites harboring the NNGGGT PAM [36]. In contrast to SpCas9, we found that SaCas9 is more sensitive to spacer length, and shorter or longer than 21–22 nt significantly decreased cutting efficiency. In addition, the 22-nt spacer showed considerably lower off-target activities than the 21-nt version. Considering both efficacy and adverse effects, we recommend using 22-nt sgRNA, such as gN21 or GN21, for SaCas9-based applications. However, screening spacers of 21, 22, and 23 nt will benefit clinical applications.

In contrast to early studies using the T7E1 mismatch cleavage assay to assess indel frequencies [14], we conducted deep sequencing to analyze cleavage outcomes accurately. For the first time, we identified a distinctive feature of SaCas9, a considerably lower propensity for the staggered cut. It is well established that SpCas9 has a strong bias for inducing staggered 5′ end overhangs after cleavage, thus resulting in a high probability of +1 nucleotide insertion [11], [12]. However, SaCas9 is ∼ 10 times less likely to generate staggered dsDNA ends. Since +1 NHEJ repair after a staggered cut occurs much faster than other repair pathways, such as HDR [12], this distinctive feature translates into a greater proportion of dsODN insertion and HDR knock-in after SaCas9 cleavage. The two Cas9 orthologs have other distinct features, *e.g.*, SpCas9 maintains binding to the DNA several hours after cleavage [37], whereas SaCas9 releases the DNA at the distal end of the PAM immediately after cleavage [38], [39]. Therefore, SaCas9 functions as a multiple-turnover enzyme, whereas SpCas9 is a single-turnover nuclease. A crystal structure comparison between SaCas9 and SpCas9 revealed notable differences in their functional domains [16]. Further investigation into the differences between SaCas9 and SpCas9 will inspire the design of novel Cas9 proteins with outstanding features.

The identified rules for optimal SaCas9, a 22-nt spacer length, and avoiding targets with the NNGGAT PAM will ensure high-level editing and minimal off-target activity. This stringency will lead to an approximately 5-fold decrease in the number of available sgRNAs compared with SpCas9, limiting its use in base editing and correction of point mutations. However, it will find applications in creating knockout phenotypes and HDR editing by targeting introns [40]. In addition, researchers have engineered SpCas9 variants, such as SpCas9-HF1, with better specificity than wild-type SpCas9 [41], [42]. Similarly, SaCas9 variants with greater specificity have also been developed [43], [44]. It would be interesting to compare the activity and fidelity of HiFi SpCas9 and HiFi SaCas9 variants in future investigations.

We observed that the optimal spacer lengths for SpCas9 and SaCas9 were 20 nt and 22 nt, respectively. However, for a few sgRNAs, a longer or shorter spacer may display greater activity. This might be explained by the sgRNA–DNA binding energy and/or sgRNA secondary structure. Similarly, SaCas9 tended to be more potent than its SpCas9 counterpart when targeting the same sequence. However, occasionally, SaCas9 is less effective than SpCas9. These observations may be attributed to the distinct effects of chromatin structure on the survey efficiency of SpCas9 and SaCas9.

In summary, we systematically described the gene-editing results of SpCas9 and SaCas9. SaCas9 cleaves the target sequence more effectively than SpCas9. Furthermore, the unique feature of SaCas9 to create blunt ends makes it more effective for dsODN insertion and HDR knock-in. Above all, SaCas9 combined with a 22-nt sgRNA showed strikingly lower off-target activities than the SpCas9 system. Our study will provide much-needed guidance for genome editing in human cells and *in vivo* gene therapy.

## Materials and methods

### Plasmid vector design and construction

We used CHOPCHOP [45] to design sgRNAs that target 11 sites on human *AAVS1*, *ALB*, *B2M1*, *B2M2*, *CIITA*, *PD1*, *TRAC*, and *CD326*. Those sgRNAs with the NGGRRT PAM were selected for simultaneously comparing two Cas9 orthologs. The sgRNA sequences are listed in Table S1. All sgRNAs were initiated with a G to ensure U6 promoter activation. The AAV HDR vectors consisted of a backbone carrying a 141-bp AAV2 inverted terminal repeat (ITR) sequence and a 6-bp short insertion flanked by 700-bp HAs.

The gene-editing experiments were conducted through plasmid electroporation. All plasmid vectors expressing Cas9, sgRNAs, or HDR donors were constructed according to our previous description [22], [46]. Briefly, all fragments were PCR amplified from human genomic DNA or existing plasmids in our lab using KAPA HiFi polymerase (Catalog No. KK2602, KAPA Biosystems, Swiss) and purified using the Zymoclean gel DNA recovery kit (Catalog No. D4001, ZYMO Research, Irvine, CA). Then the fragments were assembled into a plasmid backbone using the NEBuilder HiFi DNA assembly cloning kit (Catalog No. M5520AA, New England Biolabs, France). Multiple colonies were picked for Sanger sequencing (Tsingke Biotechnology, China) and Nanopore sequencing (GenoStarBio, China) to identify the correct clone.

### Cell culture

iPSCs used in this study were derived from human adult peripheral blood as previously described [12], [47]. Cells were cultured in mTeSR E8 medium (Catalog No. 85850, Stemcell Technologies, Canada) on Matrigel (Catalog No. 354277, BD, Becton, NJ)-coated tissue culture plates and kept in a humidified incubator at 37 °C and 5% CO_2_. The medium was daily refreshed, and the cells were routinely passaged using 1 mM EDTA after reaching 80% confluence.

K562 cells were maintained in RPMI-1640 medium (Catalog No. 12633020, Gibco, CA) supplemented with 10% (v/v) fetal bovine serum (Catalog No. 12664025, Gibco) and 1% (v/v) penicillin/streptomycin (Invitrogen, CA) in a 37 °C, 5% CO_2_, and fully humified incubator. Medium changes were usually performed 2–3 times per week.

### AAV6 packaging, purification, and titering

Recombinant AAV6 vectors were produced through a PEI (polyethyleneimine) MAX 40K (Catalog No. 24765-1, Polysciences, PA) transfection system as previously described [48]. Briefly, HEK293T (ATCC) cells at a confluency of ∼ 85% were transfected with plasmids expressing AAV6 capsid, AAV helper, and HDR donor. 5 U/ml benzonase (Catalog No. 9025654, SCBT, Dallas, TX) was added to the medium 18 h pre-harvest to eliminate the residual plasmid. Cells were treated with 500 mM NaCl (Sigma, MO) 5 days later. The supernatant was harvested 2 h later and then sterilized with a 0.22-μm filter after centrifugation. We used the Minimate (PALL) tangential flow filtration system equipped with a 300-KD molecular weight cutoff (MWCO) capsule to concentrate the supernatant. Then the AAV6 products were purified with iodixanol gradient centrifugation. The vector titer was analyzed by qPCR as described previously [48].

### dsODN preparation

The blunt-ended dsODN used in our study was prepared by annealing two modified ssODNs (5′-P-G*T*TTAATTGAGTTGTCATATGTTAATAACGGT*A*T-3′ and 5′-P- A*T*ACCGTTATTAACATATGACAACTCAATTAA*A*C-3′, where P represents 5′ phosphorylation and * indicates a phosphorothioate linkage, IDT) [47] with the following program: 95 °C for 5 min and then slowly brought to room temperature. dsODN at 50 pmol was used in each transfection.

### Plasmid electroporation

Cells were electroporated using a Lonza 2b nucleofector following the manufacturer’s recommended protocol. iPSCs at 60%–70% confluency were dissociated into a single-cell suspension and electroporated by human stem cell Nucleofector kit 2 with program B-016. 10 μM ROCK inhibitor Y27632 (Catalog No. 04001210, STEMGENT, PA) was maintained in the iPSC culture on the first day after transfection. K562 electroporation was performed with the Amaxa cell line Nucleofector kit V (Catalog No. VVCA1003, Lonza, Swiss) in program T-016.

We used 1 × 10^6^−2 × 10^6^ cells for each transfection and delivered 1 μg of Cas9 and 0.5 μg of sgRNA plasmids. For iPSCs, 0.5 μg of BCL-XL plasmid was also used for transient BCL-XL overexpression to increase cell viability [46]. For AAV6-mediated HDR, the AAV6 donor was added to the culture after electroporation without further manipulation. The multiplicity of infection (MOI) was typically 10,000–50,000. The AAV6-containing medium was replaced with a fresh culture medium 24 h later.

### Flow cytometry

The expression of mNeonGreen was evaluated by flow cytometry 72 h post nucleofection on a BD FACS Canto II flow cytometer. The FITC channel was used to determine the proportion of mNeonGreen-positive cells, which were considered as HDR knock-in cells. Electroporations without Cas9, fluorescent reporter HDR donors, or relevant sgRNAs were also performed as negative controls. The FACS data were analyzed by FlowJo v10.

### Quantification of genome-editing events

To evaluate genome-editing efficiency, we performed PCR followed by Illumina deep sequencing. In brief, approximately 1 × 10^5^ cells were harvested 72 h after electroporation for genomic DNA extraction as described previously [12]. Nested PCR was conducted to avoid HDR artifacts induced by AAV6 donors. Primers for amplifying target sequences are listed in Tables S2 and S3. DNA amplicon libraries were prepared with barcoded primers using KAPA HiFi DNA polymerase (Roche Sequencing, Swiss). Libraries were pooled and sequenced using Illumina NovaSeq6000 system (Novogene, China). The paired-end raw data were processed with Seqkit [49] and demultiplexed with Barcode-splitter (https://pypi.org/project/barcode-splitter/). The editing frequencies, HDR efficiencies, and dsODN insertion rates were analyzed and visualized using CRISPResso2 [12].

### GUIDE-seq

We conducted GUIDE-seq to investigate the off-targets following published methods [31]. We transfected cells with SaCas9 or SpCas9, the corresponding sgRNA, and the dsODN bait. Genomic DNA was extracted 72 h post-transfection, and 2 μg gDNA was used for NGS library construction following the GUIDE-seq method with minor modifications. Briefly, DNA was sheared, followed by adaptor ligation and two rounds of PCR enrichment for 34-bp dsODN baits. The PCR products were pooled for 150-bp paired-end Illumina sequencing (Novogene, china). The raw data were preprocessed with Seqkit [49] and analyzed through the GUIDE-seq software workflow. Alignments were used to identify genome-wide dsODN integration sites. Off-targets bearing up to 6 mismatches within the protospacer were identified.

### Statistical analysis and reproducibility

We conducted the data statistical analysis with GraphPad Prism 8. Significance was calculated using paired or unpaired two-tailed Student’s *t*-test for normally distributed data. Wilcoxon matched-pairs tests were conducted for the abnormally distributed data. All adjusted *P* values are indicated in the figures. *P* values of less than 0.05 were considered statistically significant. The data presented in this study were acquired from at least three independent experiments.

## Data availability

The Illumina sequencing raw data have been deposited in the Genome Sequence Archive for Human [50] at the National Genomics Data Center, Beijing Institute of Gemonics, Chinese Academy of Sciences / China National Center for Bioinformation (GSA-Human: HRA002490), and are publicly accessible at https://ngdc.cncb.ac.cn/gsa-human/.

## Competing interests

The authors have declared no competing interests.

## CRediT authorship contribution statement

**Zhi-Xue Yang:** Investigation, Methodology, Writing – original draft, Visualization. **Ya-Wen Fu:** Investigation, Formal analysis, Data curation, Writing – review & editing. **Juan-Juan Zhao:** Investigation. **Feng Zhang:** Investigation. **Si-Ang Li:** Software. **Mei Zhao:** Investigation. **Wei Wen:** Investigation. **Lei Zhang:** Funding acquisition. **Tao Cheng:** Resources, Funding acquisition. **Jian-Ping Zhang:** Writing – review & editing, Funding acquisition. **Xiao-Bing Zhang:** Conceptualization, Supervision, Project administration, Writing – review & editing, Funding acquisition. All authors have read and approved the final manuscript.

## Supplementary material

Supplementary Figure S1 **Reproducible genome editing results in iPSCs and K562 cells A.** The reproducibility of indel data at 48 h after electroporation of iPSCs with SaCas9-sgRNA plasmids. **B.** The reproducibility of indel data at 72 h after electroporation. We conducted Pearson linear regression analysis on indel data from two biological replicates. **C.** Almost identical indel frequencies were assessed 48 h and 72 h after electroporation in iPSCs. **D.** The reproducibility of indel data at 48 h after electroporation of K562 cells with SaCas9-sgRNA plasmids. **E.** The reproducibility of indel data at 72 h after electroporation of K562 cells. Pearson linear regression analysis was conducted on indel data from two biological replicates. **F.** Almost identical indel frequencies were assessed at 48 h and 72 h after electroporation in K562 cells. iPSCs, human induced pluripotent stem cells.

Supplementary Figure S2 **Raw values of indel frequencies after editing with SpCas9 and sgRNAs of 18-21 nt** The sgRNA sequences are listed below each panel. Data are shown as mean ± s.d. (n = 3–4 for each). Indel, insertion and deletion.

Supplementary Figure S3 **Raw values of indel frequencies after editing with SaCas9 and sgRNA of 19-23 nt** The sgRNA sequences are listed below each panel. Data are shown as mean ± s.d. (n = 4 for each).

Supplementary Figure S4 **Superior editing potency of SaCas9 to SaCas9 in both iPSCs and K562 cells** The data presented in Figure 5B were reanalyzed by excluding the low-performance sgRNAs targeting the NNGGAT PAM. All the indel values were normalized to the average editing efficiency of SpCas9. Data are shown as mean ± s.d. Significance was calculated using an unpaired two-tailed Student’s *t*-test. PAM, protospacer adjacent motif.

Supplementary Figure S5 **Repair patterns of all 11 sites in both iPSCs and K562 cells** The reference target sequence is shown on the top line. The Cas9 cleavage site is labeled with a vertical dotted line. The nucleotide insertion events are shown in the red box, and deletions are displayed by horizontal dotted lines. The frequencies and read counts corresponding to each allele are shown on the right.

Supplementary Figure S6 **The SpCas9 off-targets of sg*AAVS1c* identified by GUIDE-seq** The intended target sequence with PAM is shown on the top line. Mismatches found in off-target sequences are highlighted in color. The read counts corresponding to different spacer lengths are shown on the right.

Supplementary Table S1 **Comparison of the gene-editing results between SpCas9 and SaCas9 in K562 cells**

Supplementary Table S2 **1st PCR primers for assessing AAV KI frequencies**

Supplementary Table S3 **The primers for assessing KO frequencies or AAV HDR frequencies**
