# Supplementary material for: Superior Fidelity and Distinct Editing Outcomes of SaCas9 Compared with SpCas9 in Genome Editing
Source: Genomics Proteomics Bioinformatics. 2022 Dec 20;21(6):1206–20. doi: 10.1016/j.gpb.2022.12.003 (PMC11082263; doi:10.1016/j.gpb.2022.12.003)
Supplement: Supplementary Figure S1 — Reproducible genome editing results in iPSCs and K562 cells A. The reproducibility of indel data at 48 h after electroporation of iPSCs with SaCas9-sgRNA plasmids. B. The reproducibility of indel data at 72 h after electroporation. We conducted Pearson linear regression analysis on indel data from two biological replicates. C. Almost identical indel frequencies were assessed 48 h and 72 h after electroporation in iPSCs. D. The reproducibility of indel data at 48 h after electroporation of K562 cells with SaCas9-sgRNA plasmids. E. The reproducibility of indel data at 72 h after electroporation of K562 cells. Pearson linear regression analysis was conducted on indel data from two biological replicates. F. Almost identical indel frequencies were assessed at 48 h and 72 h after electroporation in K562 cells. iPSCs, human induced pluripotent stem cells. [file mmc1.pptx]

## Slide 1
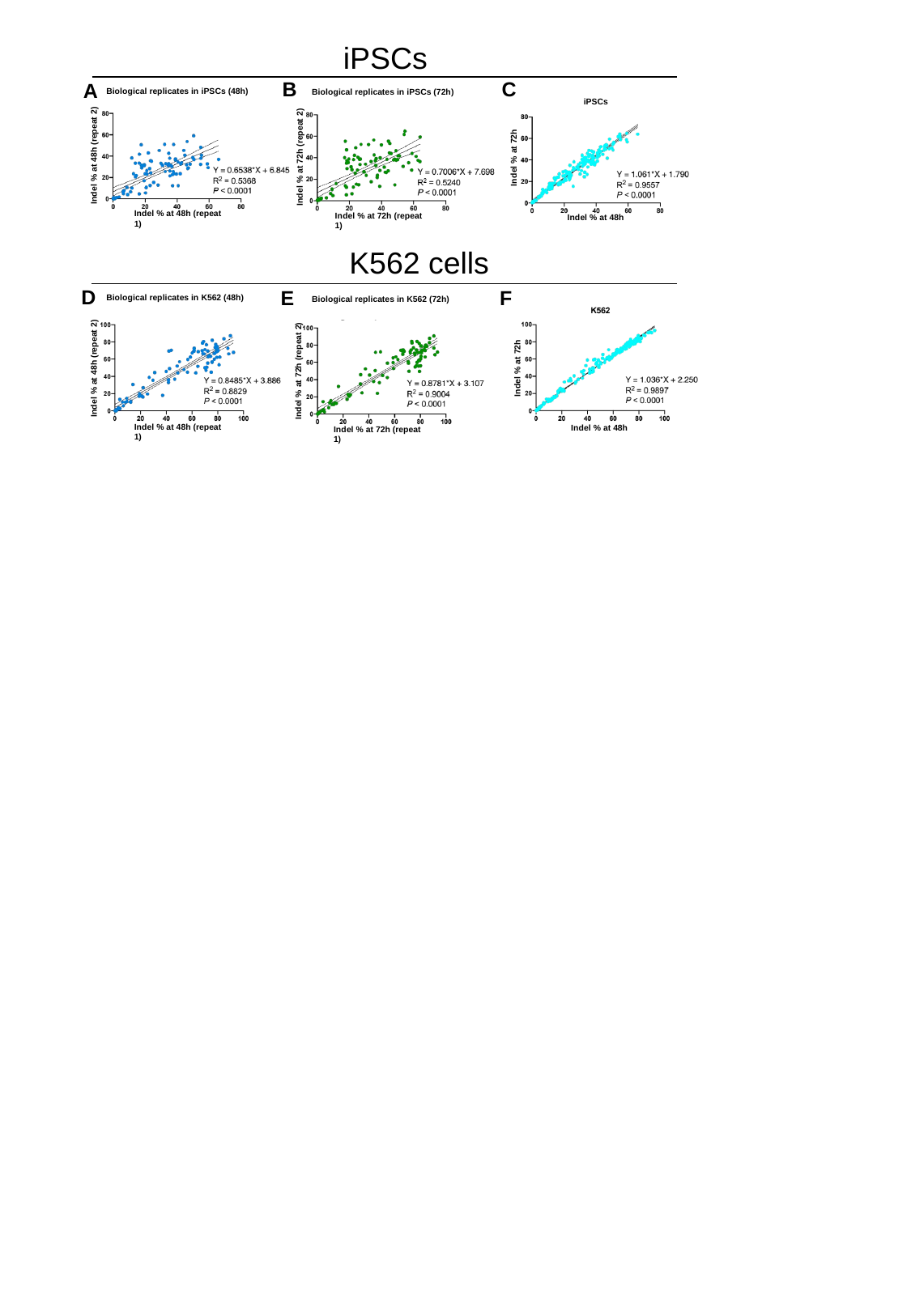

iPSCs
B
C
A
Biological replicates in iPSCs (48h)
Biological replicates in iPSCs (72h)
iPSCs
Indel % at 48h (repeat 2)
Indel % at 72h (repeat 2)
Indel % at 72h
Indel % at 48h (repeat 1)
Indel % at 72h (repeat 1)
Indel % at 48h
K562 cells
D
E
F
Biological replicates in K562 (48h)
Biological replicates in K562 (72h)
Indel % at 48h (repeat 2)
Indel % at 72h
Indel % at 72h (repeat 2)
Indel % at 48h (repeat 1)
Indel % at 48h
Indel % at 72h (repeat 1)
