# Supplementary material for: Superior Fidelity and Distinct Editing Outcomes of SaCas9 Compared with SpCas9 in Genome Editing
Source: Genomics Proteomics Bioinformatics. 2022 Dec 20;21(6):1206–20. doi: 10.1016/j.gpb.2022.12.003 (PMC11082263; doi:10.1016/j.gpb.2022.12.003)
Supplement: Supplementary Figure S2 — Raw values of indel frequencies after editing with SpCas9 and sgRNAs of 18-21 nt The sgRNA sequences are listed below each panel. Data are shown as mean ± s.d. (n = 3–4 for each). Indel, insertion and deletion. [file mmc2.pptx]

## Slide 1
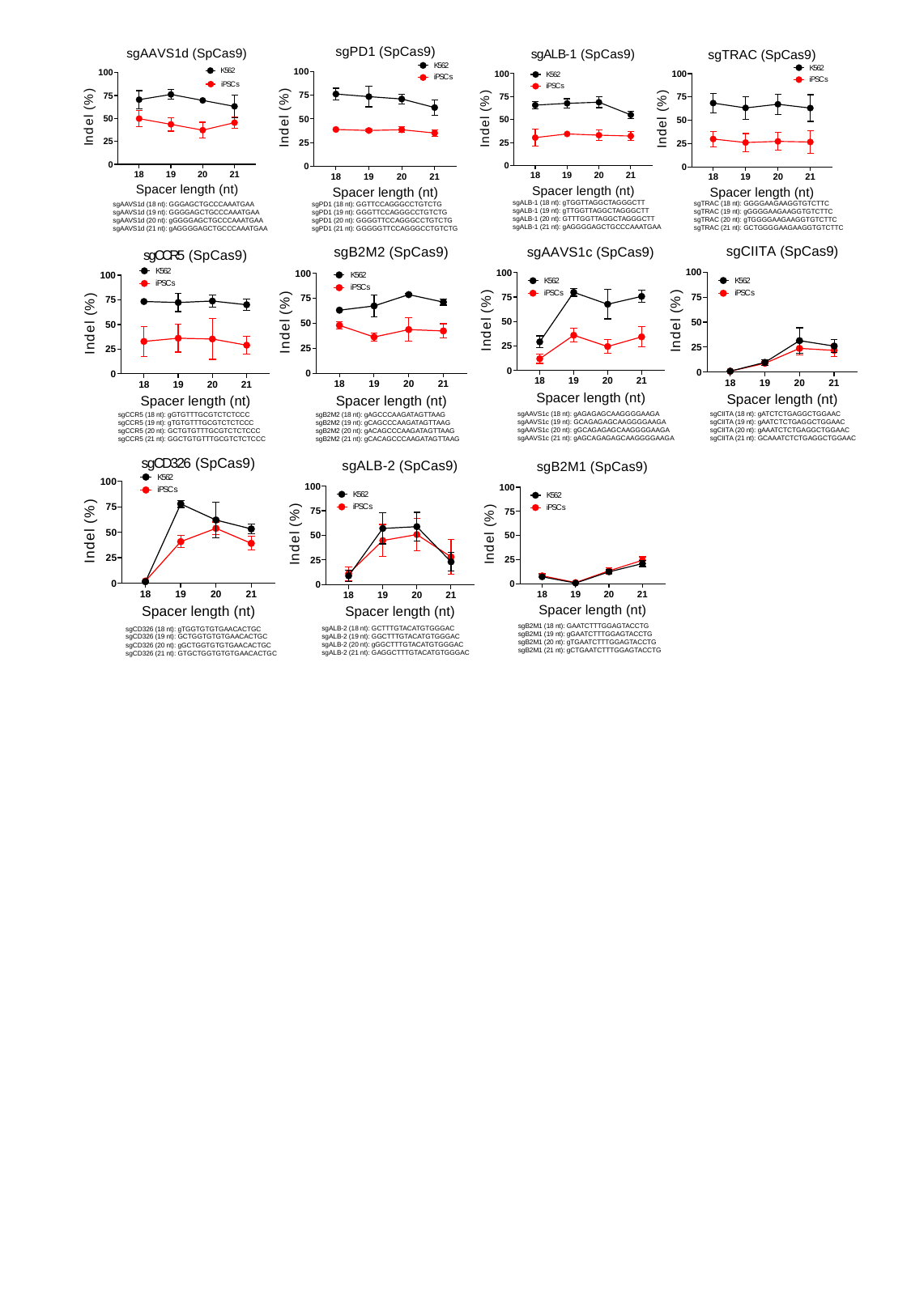

sgALB-1 (18 nt): gTGGTTAGGCTAGGGCTT
sgALB-1 (19 nt): gTTGGTTAGGCTAGGGCTT
sgALB-1 (20 nt): GTTTGGTTAGGCTAGGGCTT
sgALB-1 (21 nt): gAGGGGAGCTGCCCAAATGAA
sgTRAC (18 nt): GGGGAAGAAGGTGTCTTC
sgTRAC (19 nt): gGGGGAAGAAGGTGTCTTC
sgTRAC (20 nt): gTGGGGAAGAAGGTGTCTTC
sgTRAC (21 nt): GCTGGGGAAGAAGGTGTCTTC
sgPD1 (18 nt): GGTTCCAGGGCCTGTCTG
sgPD1 (19 nt): GGGTTCCAGGGCCTGTCTG
sgPD1 (20 nt): GGGGTTCCAGGGCCTGTCTG
sgPD1 (21 nt): GGGGGTTCCAGGGCCTGTCTG
sgAAVS1d (18 nt): GGGAGCTGCCCAAATGAA
sgAAVS1d (19 nt): GGGGAGCTGCCCAAATGAA
sgAAVS1d (20 nt): gGGGGAGCTGCCCAAATGAA
sgAAVS1d (21 nt): gAGGGGAGCTGCCCAAATGAA
sgCIITA (18 nt): gATCTCTGAGGCTGGAAC
sgCIITA (19 nt): gAATCTCTGAGGCTGGAAC
sgCIITA (20 nt): gAAATCTCTGAGGCTGGAAC
sgCIITA (21 nt): GCAAATCTCTGAGGCTGGAAC
sgAAVS1c (18 nt): gAGAGAGCAAGGGGAAGA
sgAAVS1c (19 nt): GCAGAGAGCAAGGGGAAGA
sgAAVS1c (20 nt): gGCAGAGAGCAAGGGGAAGA
sgAAVS1c (21 nt): gAGCAGAGAGCAAGGGGAAGA
sgCCR5 (18 nt): gGTGTTTGCGTCTCTCCC
sgCCR5 (19 nt): gTGTGTTTGCGTCTCTCCC
sgCCR5 (20 nt): GCTGTGTTTGCGTCTCTCCC
sgCCR5 (21 nt): GGCTGTGTTTGCGTCTCTCCC
sgB2M2 (18 nt): gAGCCCAAGATAGTTAAG
sgB2M2 (19 nt): gCAGCCCAAGATAGTTAAG
sgB2M2 (20 nt): gACAGCCCAAGATAGTTAAG
sgB2M2 (21 nt): gCACAGCCCAAGATAGTTAAG
sgB2M1 (18 nt): GAATCTTTGGAGTACCTG
sgB2M1 (19 nt): gGAATCTTTGGAGTACCTG
sgB2M1 (20 nt): gTGAATCTTTGGAGTACCTG
sgB2M1 (21 nt): gCTGAATCTTTGGAGTACCTG
sgALB-2 (18 nt): GCTTTGTACATGTGGGAC
sgALB-2 (19 nt): GGCTTTGTACATGTGGGAC
sgALB-2 (20 nt): gGGCTTTGTACATGTGGGAC
sgALB-2 (21 nt): GAGGCTTTGTACATGTGGGAC
sgCD326 (18 nt): gTGGTGTGTGAACACTGC
sgCD326 (19 nt): GCTGGTGTGTGAACACTGC
sgCD326 (20 nt): gGCTGGTGTGTGAACACTGC
sgCD326 (21 nt): GTGCTGGTGTGTGAACACTGC
