# Supplementary material for: Superior Fidelity and Distinct Editing Outcomes of SaCas9 Compared with SpCas9 in Genome Editing
Source: Genomics Proteomics Bioinformatics. 2022 Dec 20;21(6):1206–20. doi: 10.1016/j.gpb.2022.12.003 (PMC11082263; doi:10.1016/j.gpb.2022.12.003)
Supplement: Supplementary Figure S3 — Raw values of indel frequencies after editing with SaCas9 and sgRNA of 19-23 nt The sgRNA sequences are listed below each panel. Data are shown as mean ± s.d. (n = 4 for each). [file mmc3.pptx]

## Slide 1
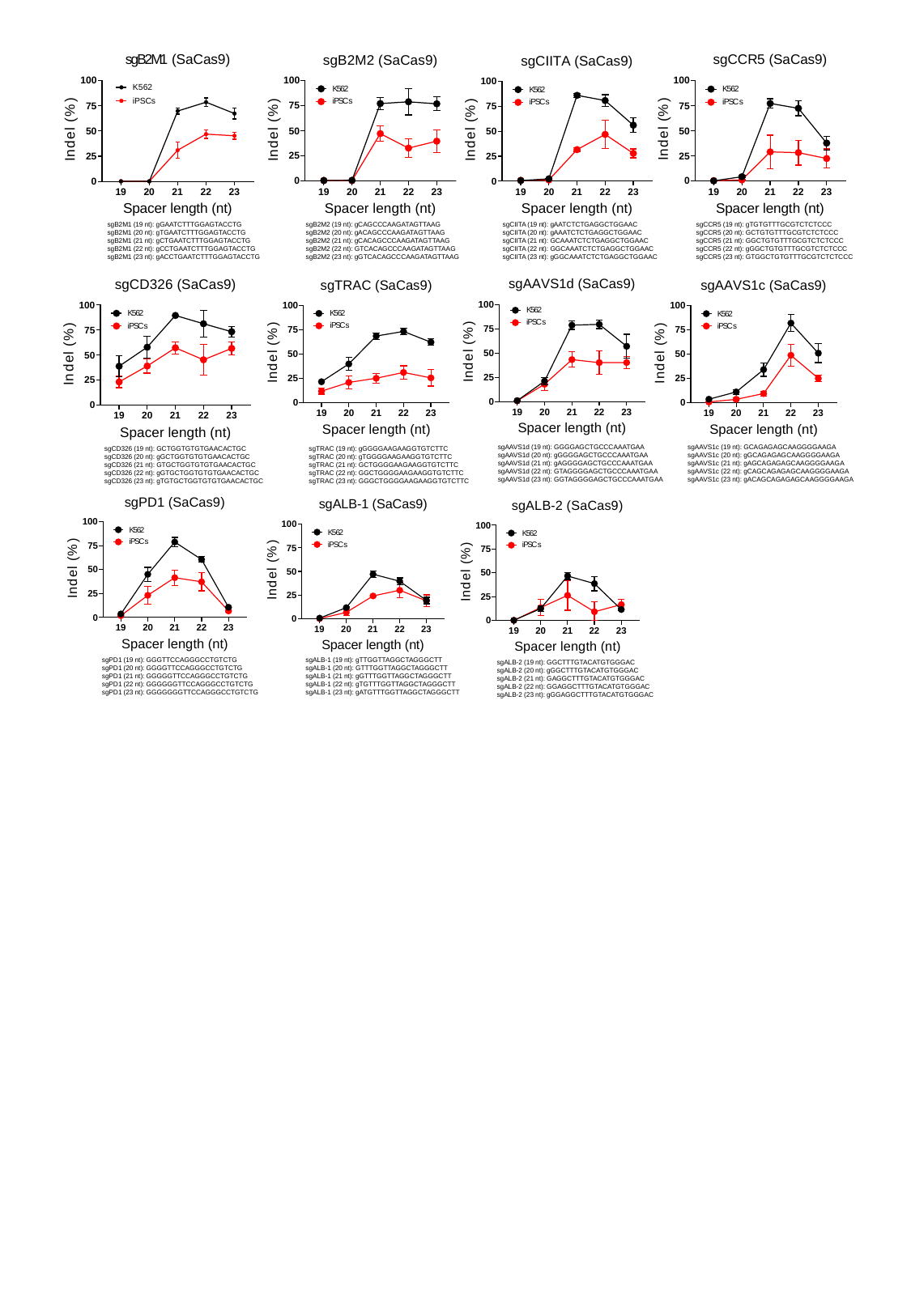

sgB2M2 (19 nt): gCAGCCCAAGATAGTTAAG
sgB2M2 (20 nt): gACAGCCCAAGATAGTTAAG
sgB2M2 (21 nt): gCACAGCCCAAGATAGTTAAG
sgB2M2 (22 nt): GTCACAGCCCAAGATAGTTAAG
sgB2M2 (23 nt): gGTCACAGCCCAAGATAGTTAAG
sgCIITA (19 nt): gAATCTCTGAGGCTGGAAC
sgCIITA (20 nt): gAAATCTCTGAGGCTGGAAC
sgCIITA (21 nt): GCAAATCTCTGAGGCTGGAAC
sgCIITA (22 nt): GGCAAATCTCTGAGGCTGGAAC
sgCIITA (23 nt): gGGCAAATCTCTGAGGCTGGAAC
sgCCR5 (19 nt): gTGTGTTTGCGTCTCTCCC
sgCCR5 (20 nt): GCTGTGTTTGCGTCTCTCCC
sgCCR5 (21 nt): GGCTGTGTTTGCGTCTCTCCC
sgCCR5 (22 nt): gGGCTGTGTTTGCGTCTCTCCC
sgCCR5 (23 nt): GTGGCTGTGTTTGCGTCTCTCCC
sgB2M1 (19 nt): gGAATCTTTGGAGTACCTG
sgB2M1 (20 nt): gTGAATCTTTGGAGTACCTG
sgB2M1 (21 nt): gCTGAATCTTTGGAGTACCTG
sgB2M1 (22 nt): gCCTGAATCTTTGGAGTACCTG
sgB2M1 (23 nt): gACCTGAATCTTTGGAGTACCTG
sgAAVS1d (19 nt): GGGGAGCTGCCCAAATGAA
sgAAVS1d (20 nt): gGGGGAGCTGCCCAAATGAA
sgAAVS1d (21 nt): gAGGGGAGCTGCCCAAATGAA
sgAAVS1d (22 nt): GTAGGGGAGCTGCCCAAATGAA
sgAAVS1d (23 nt): GGTAGGGGAGCTGCCCAAATGAA
sgAAVS1c (19 nt): GCAGAGAGCAAGGGGAAGA
sgAAVS1c (20 nt): gGCAGAGAGCAAGGGGAAGA
sgAAVS1c (21 nt): gAGCAGAGAGCAAGGGGAAGA
sgAAVS1c (22 nt): gCAGCAGAGAGCAAGGGGAAGA
sgAAVS1c (23 nt): gACAGCAGAGAGCAAGGGGAAGA
sgCD326 (19 nt): GCTGGTGTGTGAACACTGC
sgCD326 (20 nt): gGCTGGTGTGTGAACACTGC
sgCD326 (21 nt): GTGCTGGTGTGTGAACACTGC
sgCD326 (22 nt): gGTGCTGGTGTGTGAACACTGC
sgCD326 (23 nt): gTGTGCTGGTGTGTGAACACTGC
sgTRAC (19 nt): gGGGGAAGAAGGTGTCTTC
sgTRAC (20 nt): gTGGGGAAGAAGGTGTCTTC
sgTRAC (21 nt): GCTGGGGAAGAAGGTGTCTTC
sgTRAC (22 nt): GGCTGGGGAAGAAGGTGTCTTC
sgTRAC (23 nt): GGGCTGGGGAAGAAGGTGTCTTC
sgPD1 (19 nt): GGGTTCCAGGGCCTGTCTG
sgPD1 (20 nt): GGGGTTCCAGGGCCTGTCTG
sgPD1 (21 nt): GGGGGTTCCAGGGCCTGTCTG
sgPD1 (22 nt): GGGGGGTTCCAGGGCCTGTCTG
sgPD1 (23 nt): GGGGGGGTTCCAGGGCCTGTCTG
sgALB-1 (19 nt): gTTGGTTAGGCTAGGGCTT
sgALB-1 (20 nt): GTTTGGTTAGGCTAGGGCTT
sgALB-1 (21 nt): gGTTTGGTTAGGCTAGGGCTT
sgALB-1 (22 nt): gTGTTTGGTTAGGCTAGGGCTT
sgALB-1 (23 nt): gATGTTTGGTTAGGCTAGGGCTT
sgALB-2 (19 nt): GGCTTTGTACATGTGGGAC
sgALB-2 (20 nt): gGGCTTTGTACATGTGGGAC
sgALB-2 (21 nt): GAGGCTTTGTACATGTGGGAC
sgALB-2 (22 nt): GGAGGCTTTGTACATGTGGGAC
sgALB-2 (23 nt): gGGAGGCTTTGTACATGTGGGAC
