# Supplementary figures and images for: Superior Fidelity and Distinct Editing Outcomes of SaCas9 Compared with SpCas9 in Genome Editing
Source: Genomics Proteomics Bioinformatics. 2022 Dec 20;21(6):1206–20. doi: 10.1016/j.gpb.2022.12.003 (PMC11082263; doi:10.1016/j.gpb.2022.12.003)

## Slide 1
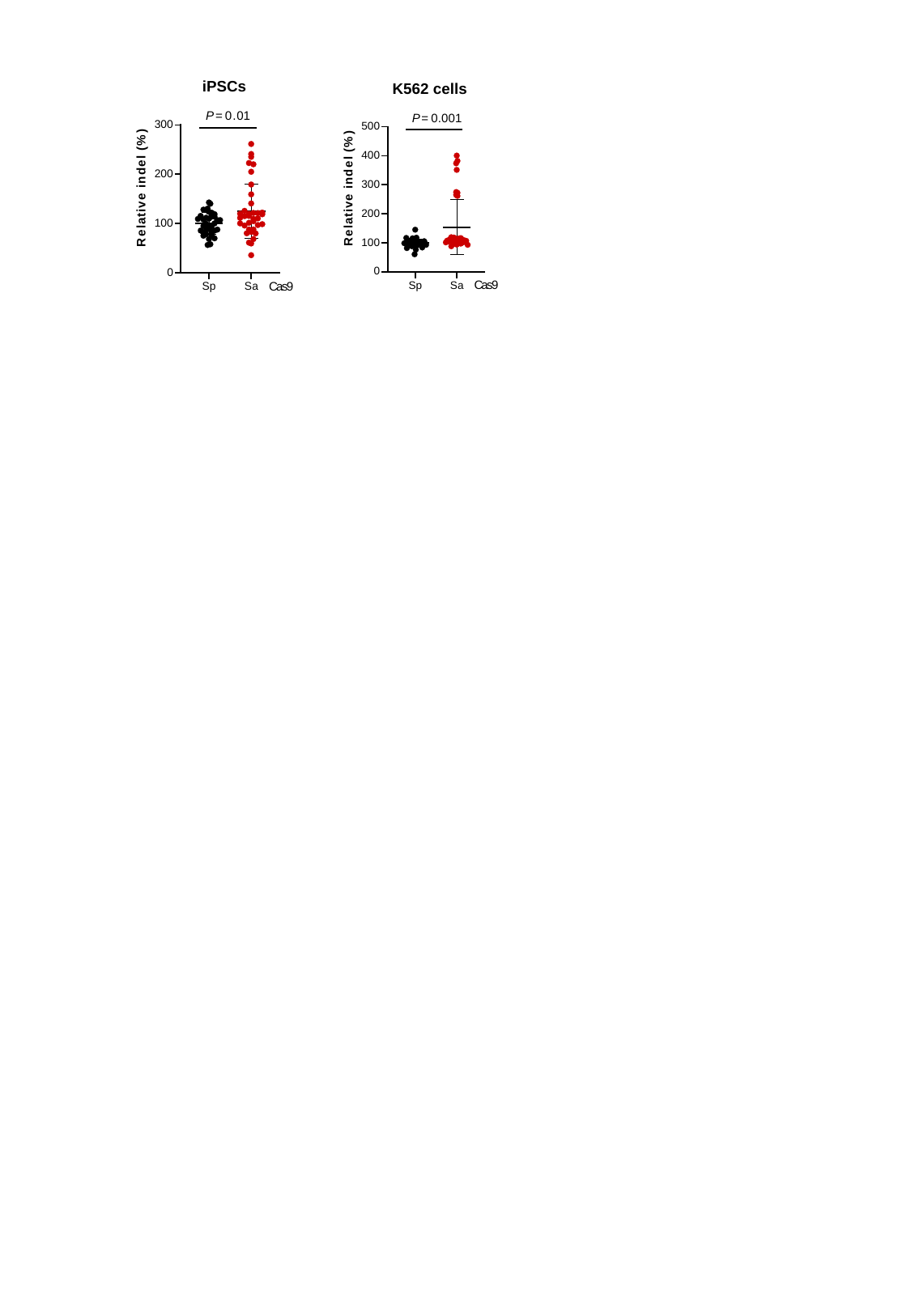

iPSCs
K562 cells

Supplement: Supplementary Figure S4 — Superior editing potency of SaCas9 to SaCas9 in both iPSCs and K562 cells The data presented in Figure 5B were reanalyzed by excluding the low-performance sgRNAs targeting the NNGGAT PAM. All the indel values were normalized to the average editing efficiency of SpCas9. Data are shown as mean ± s.d. Significance was calculated using an unpaired two-tailed Student’s t-test. PAM, protospacer adjacent motif. [file mmc4.pptx]

## Slide 1
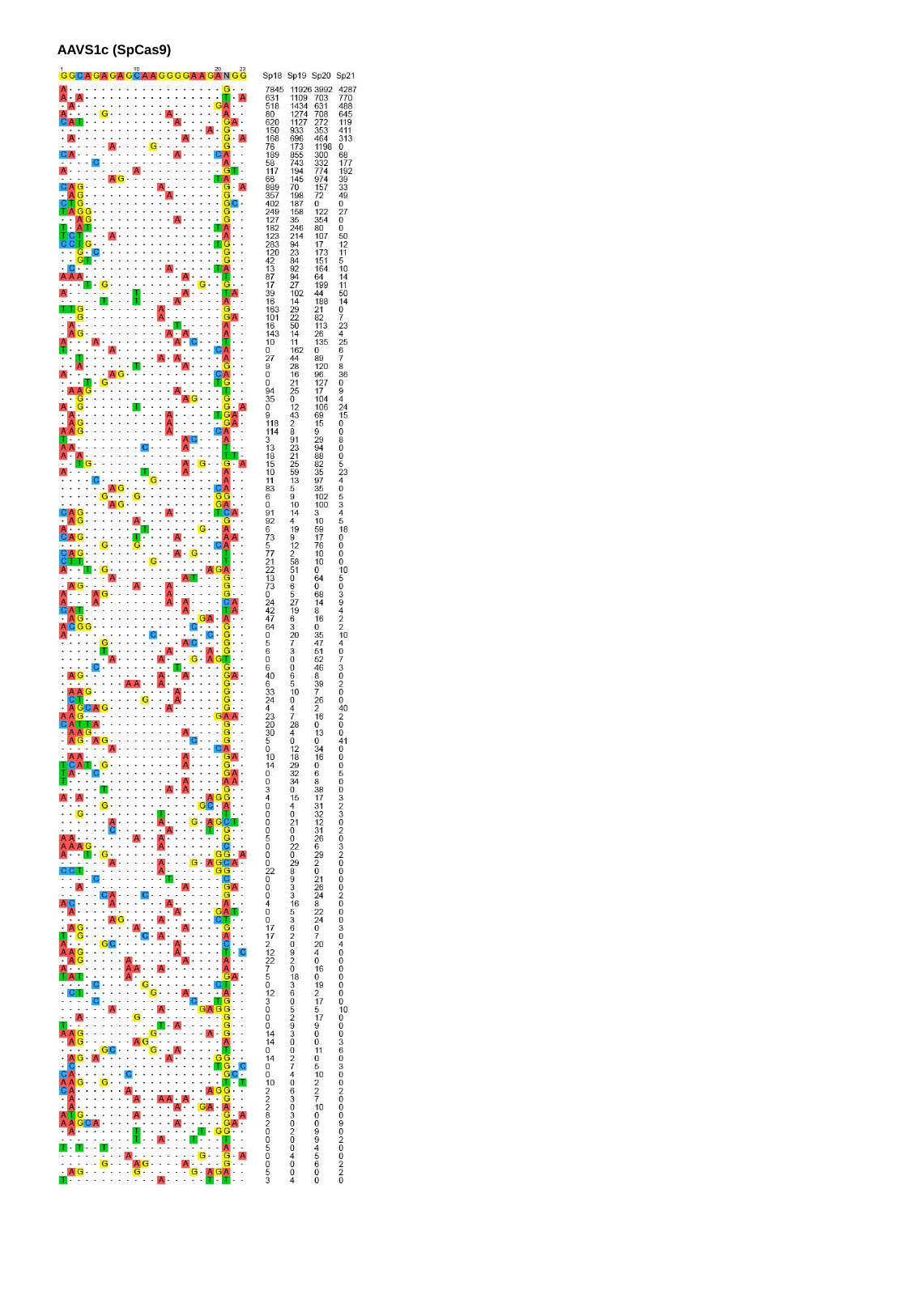

AAVS1c (SpCas9)

Supplement: Supplementary Figure S6 — The SpCas9 off-targets of sgAAVS1c identified by GUIDE-seq The intended target sequence with PAM is shown on the top line. Mismatches found in off-target sequences are highlighted in color. The read counts corresponding to different spacer lengths are shown on the right. [file mmc6.pptx]
