# Supplementary material for: Superior Fidelity and Distinct Editing Outcomes of SaCas9 Compared with SpCas9 in Genome Editing
Source: Genomics Proteomics Bioinformatics. 2022 Dec 20;21(6):1206–20. doi: 10.1016/j.gpb.2022.12.003 (PMC11082263; doi:10.1016/j.gpb.2022.12.003)
Supplement: Supplementary Figure S5 — Repair patterns of all 11 sites in both iPSCs and K562 cells The reference target sequence is shown on the top line. The Cas9 cleavage site is labeled with a vertical dotted line. The nucleotide insertion events are shown in the red box, and deletions are displayed by horizontal dotted lines. The frequencies and read counts corresponding to each allele are shown on the right. [file mmc5.pptx]

## Slide 1
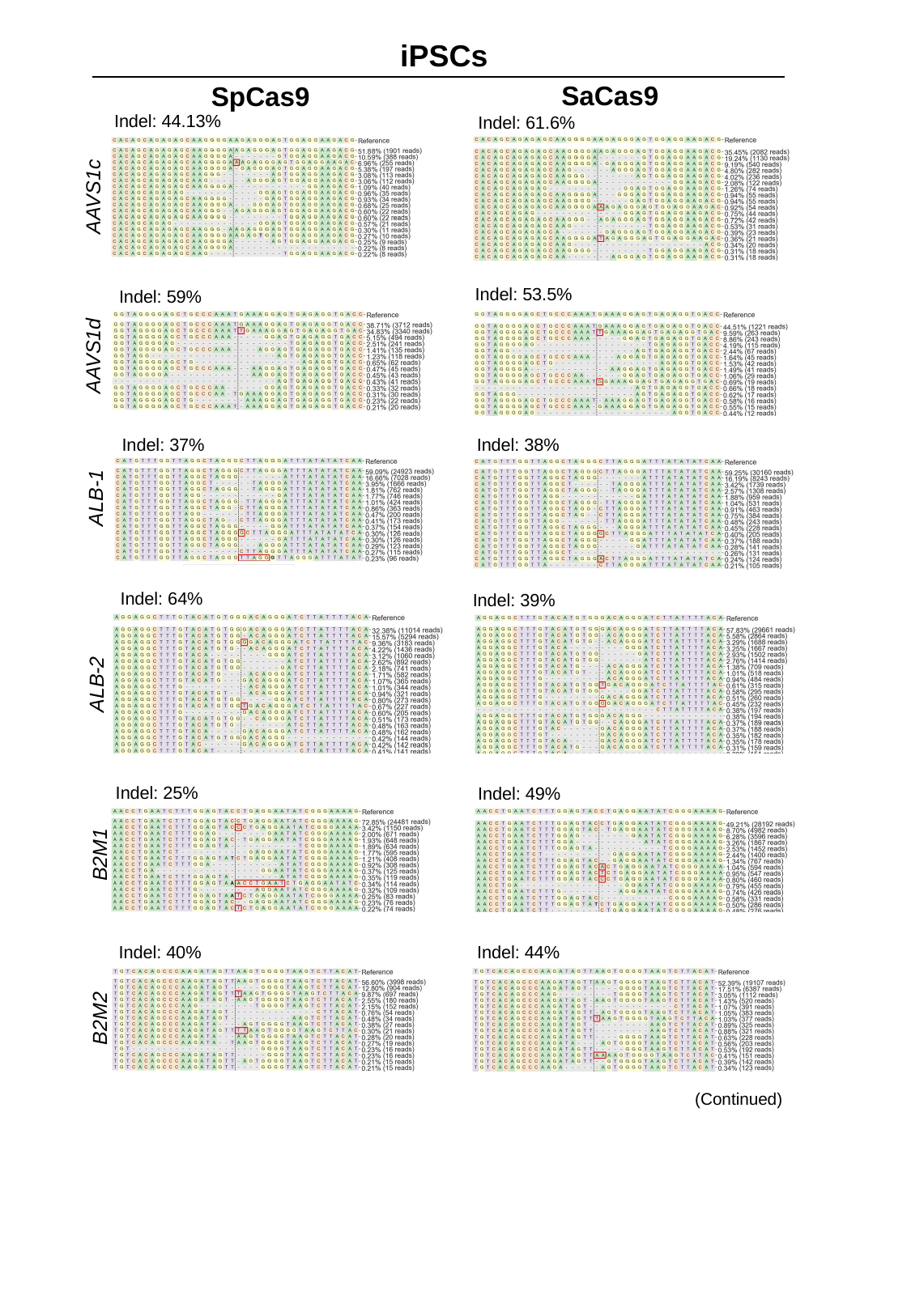

iPSCs
SaCas9
SpCas9
Indel: 44.13%
Indel: 61.6%
AAVS1c
Indel: 53.5%
Indel: 59%
AAVS1d
Indel: 38%
Indel: 37%
ALB-1
Indel: 64%
Indel: 39%
ALB-2
Indel: 25%
Indel: 49%
B2M1
Indel: 40%
Indel: 44%
B2M2
(Continued)

## Slide 2
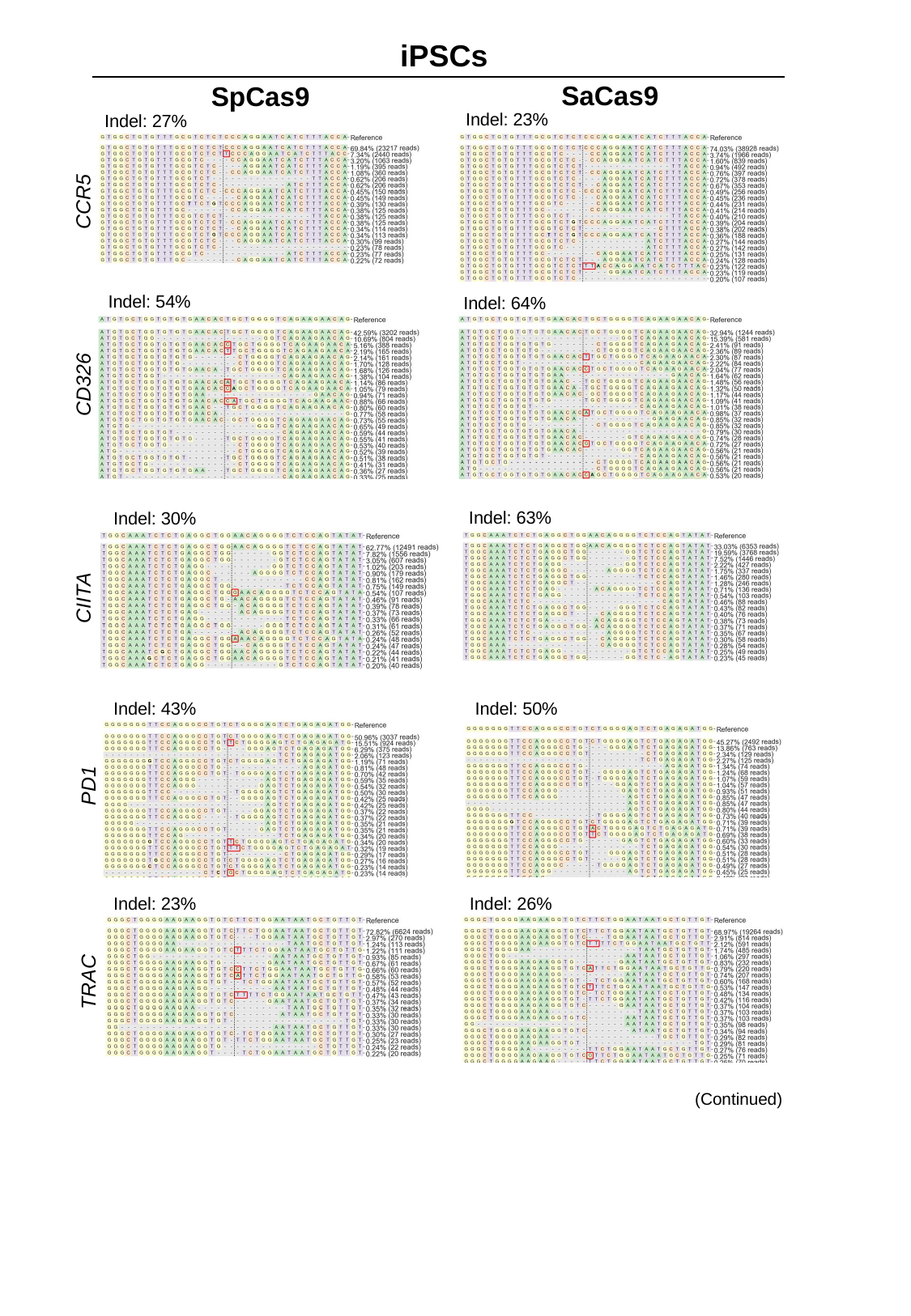

iPSCs
SaCas9
SpCas9
Indel: 23%
Indel: 27%
CCR5
Indel: 54%
Indel: 64%
CD326
Indel: 63%
Indel: 30%
CIITA
Indel: 43%
Indel: 50%
PD1
Indel: 23%
Indel: 26%
TRAC
(Continued)

## Slide 3
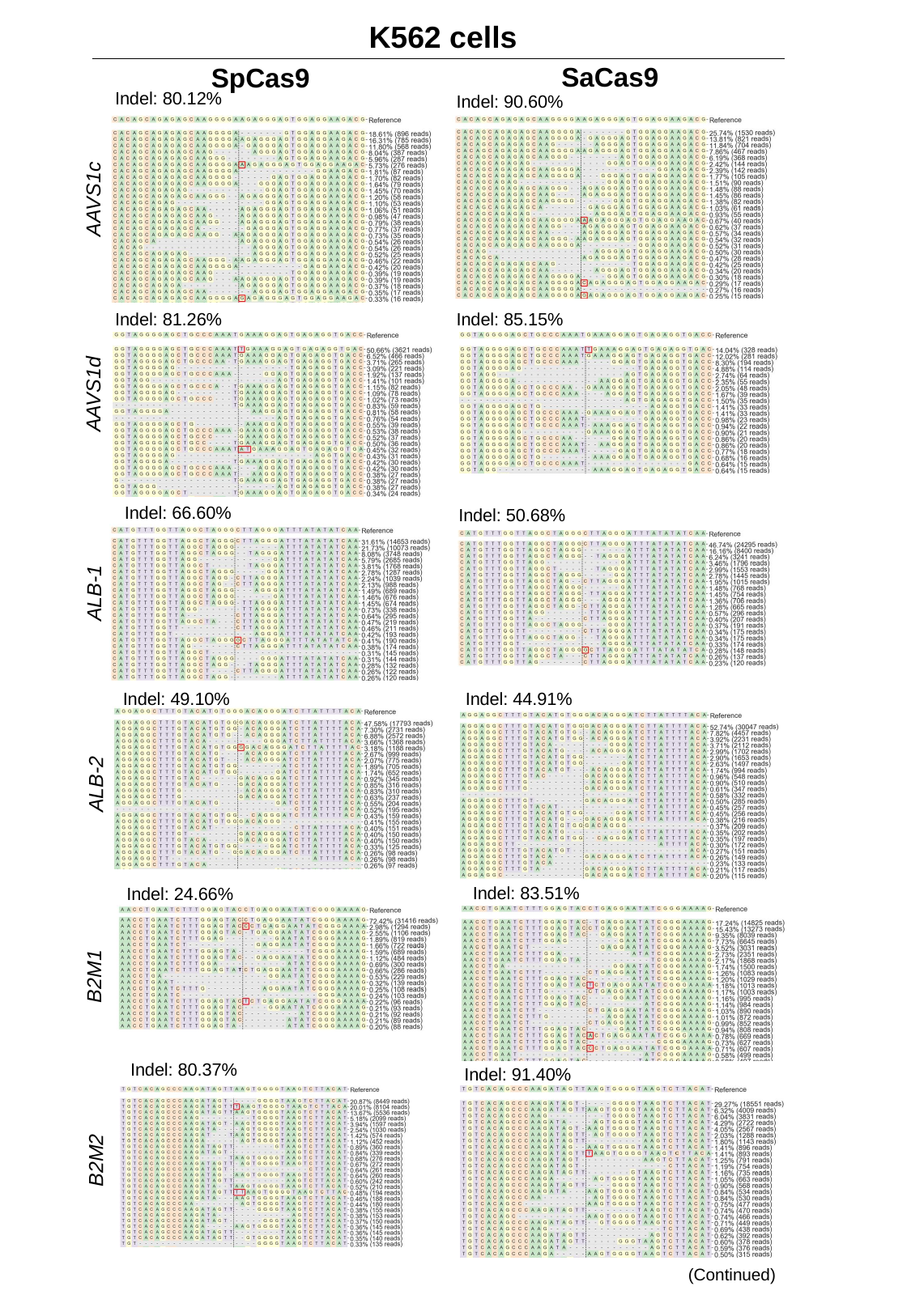

K562 cells
SaCas9
SpCas9
Indel: 80.12%
Indel: 90.60%
AAVS1c
Indel: 81.26%
Indel: 85.15%
AAVS1d
Indel: 66.60%
Indel: 50.68%
ALB-1
Indel: 49.10%
Indel: 44.91%
ALB-2
Indel: 83.51%
Indel: 24.66%
B2M1
Indel: 80.37%
Indel: 91.40%
B2M2
(Continued)

## Slide 4
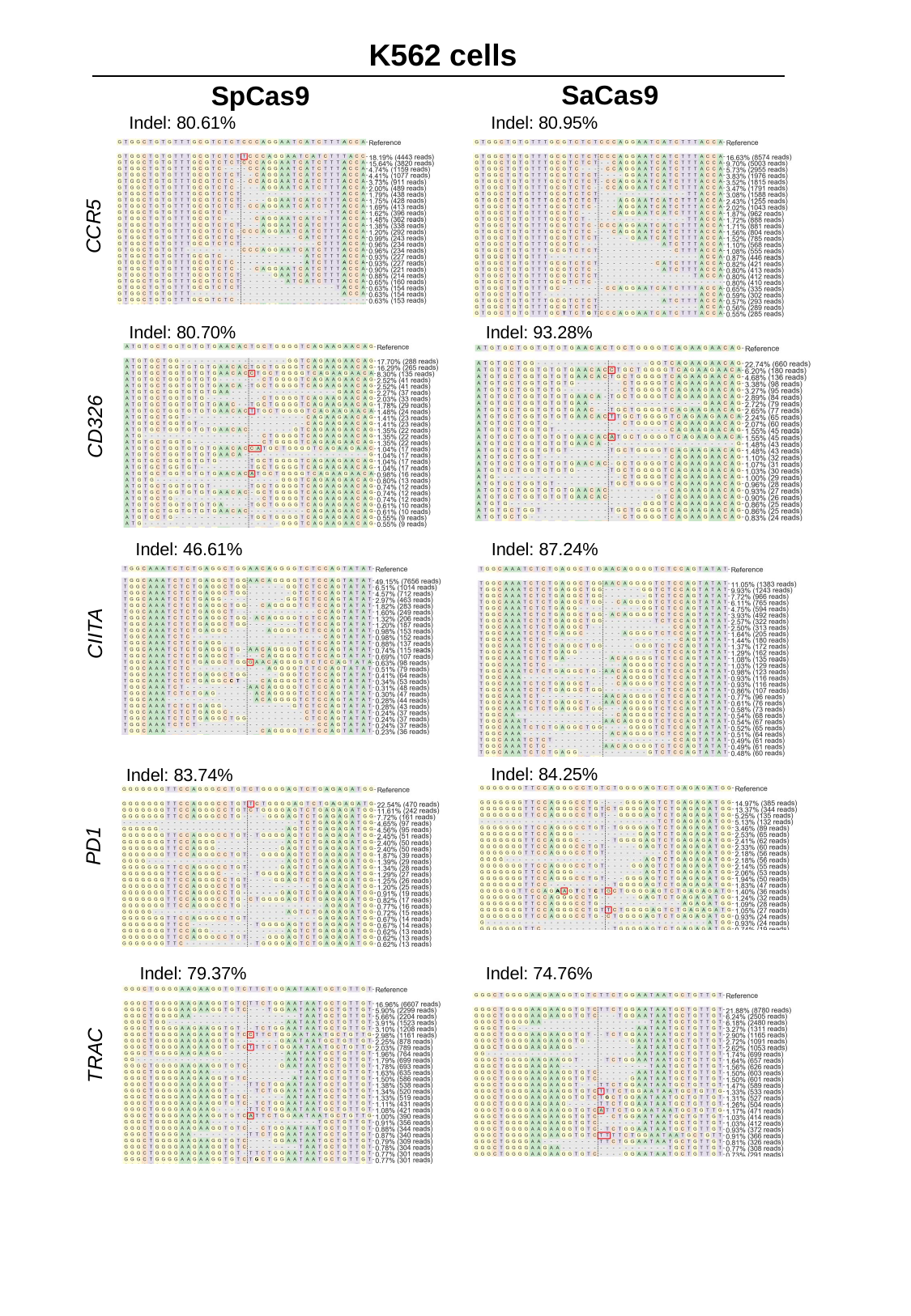

K562 cells
SaCas9
SpCas9
Indel: 80.61%
Indel: 80.95%
CCR5
Indel: 80.70%
Indel: 93.28%
CD326
Indel: 46.61%
Indel: 87.24%
CIITA
Indel: 84.25%
Indel: 83.74%
PD1
Indel: 79.37%
Indel: 74.76%
TRAC
