# Supplementary material for: Superior Fidelity and Distinct Editing Outcomes of SaCas9 Compared with SpCas9 in Genome Editing
Source: Genomics Proteomics Bioinformatics. 2022 Dec 20;21(6):1206–20. doi: 10.1016/j.gpb.2022.12.003 (PMC11082263; doi:10.1016/j.gpb.2022.12.003)
Supplement: Supplementary Table S1 — Comparison of the gene-editing results between SpCas9 and SaCas9 in K562 cells [file mmc7.docx]

**Table S1 Comparison of the gene-editing results between SpCas9 and SaCas9 in K562 cells**

| **K562** | **Target sequence (5’→3’)** | **PAM (NGGRRT)** | **Indel** | | **NHEJ +1** | | | **NHEJ -1** | | | **MMEJ** | | |
| --- | --- | --- | --- | --- | --- | --- | --- | --- | --- | --- | --- | --- | --- |
|  |  |  | **SpCas9** | **SaCas9** | **Pattern** | **SpCas9** | **SaCas9** | **Pattern** | **SpCas9** | **SaCas9** | **Pattern** | **SpCas9** | **SaCas9** |
| *AAVS1c* | CACAGCAGAGAGCAAGGGGA\|AGA | ggGAGT | 80.1% | 90.6% | +A (A\|A) | 5.7% | 0.7% | -A (A\|A) | 11.8% | 25.7% | -7 (GGGA\|AGAGGGA) | 18.6% | 13.8% |
| *AAVS1d* | GGTAGGGGAGCTGCCCAAAT\|GAA | agGAGT | 81.3% | 85.2% | +T (T\|G) | 50.7% | 14.0% | -G (T\|G) | < 0.2% | 0.9% | -5 (AAAT\|GAAA) | 1.9% | 8.3% |
| *ALB-1* | CATGTTTGGTTAGGCTAGGG\|CTT | agGGAT | 66.6% | 50.7% | +G (G\|C) | 0.4% | 0.3% | -C (G\|C) | 1.5% | 1.5% | -7 (AGGG\|CTTAGGG) | 21.7% | 16.2% |
| *ALB-2* | AGGAGGCTTTGTACATGTGG\|GAC | agGGAT | 49.1% | 44.9% | +G (G\|G) | 3.2% | < 0.2% |  |  |  | -G(G\|G) | 7.3% | 3.9% |
| *B2M1* | AACCTGAATCTTTGGAGTAC\|CTG | agGAAT | 24.7% | 83.5% | +C (C\|C) | 3.0% | 0.7% |  |  |  | -C(C\|C) | 2.6% | 17.2% |
| *B2M2* | TGTCACAGCCCAAGATAGTT\|AAG | tgGGGT | 80.4% | 91.4% | +T (T\|A) | 20.0% | 1.4% | -T (T\|A) | 3.9% | 4.1% | -5 (AGTT\|AAGT) | 20.9% | 29.3% |
| *CCR5* | GTGGCTGTGTTTGCGTCTCT\|CCC | agGAAT | 80.6% | 81.0% | +T (T\|C) | 18.2% | < 0.2% | -T (T\|C) | 1.2% | 1.7% | -4 (TCTCT\|C) | 4.7% | 5.7% |
| *CD326* | ATGTGCTGGTGTGTGAACAC\|TGC | tgGGGT | 80.7% | 93.3% | +C (C\|T) | 8.3% | 6.2% | -C (C\|T) | 2.5% | 2.9% | -17 (GCTGG…\|TGCTGG) | 17.7% | 22.7% |
| *CIITA* | TGGCAAATCTCTGAGGCTGG\|AAC | agGGGT | 46.6% | 87.2% | +G (G\|A) | 0.6% | < 0.2% | -A (G\|A) | 1.3% | 3.9% | -6 (GG\|AACAGG) | 6.5% | 9.9% |
| *PD1* | GGGGGGGTTCCAGGGCCTGT\|CTG | ggGAGT | 83.7% | 84.3% | +T (T\|C) | 22.5% | 1.1% | -C(T\|C) | 2.5% | 3.5% | -4 (TGT\|CTG) | 7.7% | 15.0% |
| *TRAC* | GGGCTGGGGAAGAAGGTGTC\|TTC | tgGAAT | 79.4% | 74.8% | +C (C\|T) | 3.0% | < 0.2% | -T(C\|T) | 1.1% | 0.9% | -3 (TC\|TTC) | 5.9% | 6.2% |

*Note*: The Cas9 cleavage sites are labeled with a red vertical line. The microhomogies are highlighted with black horizontal lines. Absolute values of the indel frequency, +1 or -1 NHEJ percentage, and MMEJ frequency in K562 cells are shown. PAM, protospacer adjacent motif; NHEJ, nonhomologous end-joining; MMEJ, microhomology-mediated end-joining.
