# Supplementary material for: Superior Fidelity and Distinct Editing Outcomes of SaCas9 Compared with SpCas9 in Genome Editing
Source: Genomics Proteomics Bioinformatics. 2022 Dec 20;21(6):1206–20. doi: 10.1016/j.gpb.2022.12.003 (PMC11082263; doi:10.1016/j.gpb.2022.12.003)
Supplement: Supplementary Table S2 — 1st PCR primers for assessing AAV KI frequencies [file mmc8.docx]

**Table S2 1^st^ PCR primers for assessing AAV KI frequencies**

| **1^st^ PCR primer for AAV KI** | **Sequence (5'−3')** | **Product size** |
| --- | --- | --- |
| 4528-ALB-In13-86-F | GCTGTCATCTCTTGTGGGCT |  |
| 4529-ALB-In13-86-R | CAGCTTGACTTGCAGCAACA | 1648 bp |
| 4530-B2M2-F | GAGTGCTGAGAGGGCATCAG |  |
| 4531-B2M2-R | ATACCTGGGGCCATACACCT | 1475 bp |
| 4532-PD1-F | TCTGGAAGGGCACAAAGGTC |  |
| 4533-PD1-R | GAGGTCCTTGTCTTGGGAGC | 1311 bp |
| 4534-AAVS1d-F | TCCTCTCTGGCTCCATCGT |  |
| 4535-AAVS1d-R | TCGACTTCCCCTCTTCCGAT | 1289 bp |

*Note*: PCR, polymerase Chain reaction; AAV, adeno-associated virus; KI, knock-in.
