# Supplementary material for: Superior Fidelity and Distinct Editing Outcomes of SaCas9 Compared with SpCas9 in Genome Editing
Source: Genomics Proteomics Bioinformatics. 2022 Dec 20;21(6):1206–20. doi: 10.1016/j.gpb.2022.12.003 (PMC11082263; doi:10.1016/j.gpb.2022.12.003)
Supplement: Supplementary Table S3 — The primers for assessing KO frequencies or AAV HDR frequencies [file mmc9.docx]

**Table S3 The primers for assessing KO frequencies or AAV HDR frequencies**

| **Primer name** | **Sequence (5'−3')** | **Product size** |
| --- | --- | --- |
| 4213-sgAAVS1c-150PE-F | TTGGGTGAGGGAGGAGAGAT |  |
| 4214-sgAAVS1c-150PE-R | TTTCTGTCTGCAGCTTGTGG | 236 bp |
| 4215-sgAAVS1d-150PE-F | CGGGTTGGAGGAAGAAGACT |  |
| 4216-sgAAVS1d-150PE-R | TTTTCTGGACAACCCCAAAG | 235 bp |
| 4306-sgALB-In13-46-150PE-F | GTGCACTTGTTGAGCTCGTG |  |
| 4307-sgALB-In13-46-150PE-R | CCCTGTCCCACATGTACAAA | 246 bp |
| 4209-sgALB-In13-86-150PE-F | GCAAGGCTGACGATAAGGAG |  |
| 4210-sgALB-In13-86-150PE-R | TGGCACAATAGAGCAGGAGA | 226 bp |
| 4219-sgB2M-150PE-F | TTCAATGTCGGATGGATGAA |  |
| 4220-sgB2M-150PE-R | ATTTGGCCAGAGTGGAAATG | 241 bp |
| 4308-sgB2M2-150PE-F | ACTGAATTCACCCCCACTGA |  |
| 4309-sgB2M2-150PE-R | CATTCCCTGACAATCCCAAT | 243 bp |
| 4316-sgCCR5-150PE-F | CTGTCGTCCATGCTGTGTTT |  |
| 4317-sgCCR5-150PE-R | CCAGCCCCAAGATGACTATC | 225 bp |
| 4217-sgCD326g-150PE-F | TGTTTGGTGATGAAGGCAGA |  |
| 4218-sgCD326g-150PE-R | AGTATAGGCAGCCCCACTCA | 251 bp |
| 4310-sgCIITA-150PE-F | CAAAAGCAGAATCGCAAACA |  |
| 4311-sgCIITA-150PE-R | GGTAGGAGGGAGAGGTGGTC | 252 bp |
| 4314-sgPD1-150PE-F | ACGAAGCTCTCCGATGTGTT |  |
| 4315-sgPD1-150PE-R | CCCCAGCAGAGACTTCTCAA | 253 bp |
| 4312-sgTRAC-150PE-F | GGTTTTGGTGGCAATGGATA |  |
| 4313-sgTRAC-150PE-R | GGACTTCAAGAGCAACAGTGC | 245 bp |

*Note*: KO, knock-out; HDR, homology-directed repair.
